# Health status of living kidney donors and attitude toward donation–Results from the German Living Donor Registry (SOLKID-GNR)

**DOI:** 10.3389/fmed.2026.1781270

**Published:** 2026-06-10

**Authors:** Barbara Suwelack, Sylvia Kröncke, Jeannine Wegner, Eike Bormann, Joachim Gerß, Sarah Riepenhausen, Philipp Neuhaus, Michael Storck, Martina Koch, Claudia Sommerer

**Affiliations:** 1 Transplant Nephrology, University Hospital Muenster, Muenster, Germany; 2 Department of Medical Psychology, University Medical Center Hamburg-Eppendorf, Hamburg, Germany; 3 Institute of Biostatistics and Clinical Research, University of Muenster, Muenster, Germany; 4 Institute for Medical Informatics, University of Muenster, Muenster, Germany; 5 Department of General, Visceral and Transplant Surgery, University Hospital Mainz, Mainz, Germany; 6 Department of Nephrology, University Hospital Heidelberg, Heidelberg, Germany

**Keywords:** donor health, gender differences, living kidney donation, quality of life, registry

## Abstract

**Introduction:**

Living kidney donation is considered the optimal treatment for patients with advanced kidney disease, but it carries risks for the donors. To assess these risks, data on donors' physical and mental health is needed.

**Material and methods:**

The German Living Donor Registry collects medical and psychosocial data from 32 transplant centers. The analysis provides a representative overview of the demographics, pre-operative physical and mental health, and psychosocial situation of accepted living kidney donors in Germany, accounting for gender-specific differences.

**Results:**

From January 2020 to January 2025, 1,237 individuals aged 54.5 ± 10 years (range: 20–83 years) were examined pre-operatively. Most donors were women (63%), 56% reported at least one pre-existing disease or medication, and 13.5% had an eGFR <80 ml/min. Compared to the general German population, donors showed a higher quality of life and fewer psychological impairments and fatigue symptoms. Risk factors for impaired mental quality of life and fatigue included psychosocial stress and lower resilience.

**Discussion:**

Living kidney donors in Germany do not represent a completely healthy population, but have a high quality of life. Careful, standardized medical and psychosocial evaluation is needed to identify donations with high medical or psychosocial risk and to ensure comprehensive education to protect donors.

## Introduction

Waiting times for a kidney transplant in Germany are the longest in Europe, at 8–10 years, reflecting a serious gap in healthcare (1). At the end of 2024, 10,270 patients were waiting for a kidney transplant (2), and only 1,443 kidney transplants using organs from deceased donors and 632 using living kidney donations were performed (3).

For organ recipients, living donation is the optimal therapy (4, 5). However, for living donors, nephrectomy in this case is an operation without a medical indication, but is associated with medical and psychosocial risks.

Long waiting times could put pressure on potential donors to donate even if they are not in optimal health. In Germany, the requirements for donors are deliberately high for their own protection and are legally very strict. While donors must not be put at risk beyond the risk of surgery, this is hardly possible given the potential loss of kidney function (6). Yet, international cohort studies suggest that living donation has no serious impact on donors' health (7–18).

Notably though, meaningful prospective studies are rare, and results cannot be transferred across countries due to differences in health and social systems. Studies with large sample sizes that include both medical and psychosocial data, such as quality of life, are even rarer. Furthermore, studies in the USA and Canada show a younger donor age that is associated with a better kidney function prior to donation (14–16). The data most comparable to the German situation come from the Swiss National Registry with donors of approximately the same age (18). However, they report quality of life data for only 32% of their sample. In Germany, where there are comparatively more living donors, patients have long been calling for lifelong evaluation of data on donors' medical and psychosocial health before and after donation. This gap is now being closed by the German Living Donation Registry (Safety of the Living Kidney Donor-German National Registry—SOLKID-GNR) (19).

This paper presents data from SOLKID-GNR and provides the first representative overview of the demographic characteristics, pre-operative physical and mental health, and psychosocial situation of accepted living kidney donors in Germany, accounting for gender-specific differences. Instead of focusing on postoperative changes, this analysis aims to provide a comprehensive snapshot of the donor collective prior to donation and to highlight its unique characteristics.

## Material and methods

SOLKID-GNR was funded as part of the German Federal Ministry of Education and Research's funding measure “Establishment of model registers for health services research” (BMBF funding code 01GY1906; DRKS 0023532) (19). Positive ethics votes were issued by all participating transplant centers (Ärztekammer Westfalen-Lippe 2019-732-f-s). Pre-operative data sets of donors from 32 German transplant centers in the survey period from January 2020 to January 2025 were included (Supplementary Figure S1). Demographic and clinical data were collected using questionnaires for doctors and donors. Further questions concerned decision-making and the evaluation process. The questionnaire survey took place shortly before the planned donation and after donors had been approved for donation, once the donors had given their consent. The recruitment process in centers began at different time points due to the varying lengths of time required to draw up contracts. The centers had to ask every donor who was accepted to participate in the registry. Inclusion criteria were sufficient knowledge of German, Turkish, or Russian, because validated questionnaires were only available in these languages, and written consent to participate in the registry. To evaluate the inclusion rate, centers were also asked to report the number of recruitable donors and to state reasons for non-participation.

The current analysis is based on data collected prior to donation, after donors had been approved for donation. Questionnaires were completed by donors themselves using a digital device and pseudonymised, with transplant centers having no access to donors' responses. Both measures were intended to reduce the likelihood that donors' answers would be biased by social desirability.

### Standardized psychological questionnaires

Validated questionnaires covered the following domains for both genders and were compared with age-adjusted norm values: physical and mental quality of life (SF-12) (20), depressive symptoms (PHQ-9) (21), anxiety symptoms (GAD-7) (22), somatic symptom severity (PHQ-15) (23), fatigue (MFI-20) (24, 25), impairment due to psychosocial stressors (PHQ stress module) (26), and resilience (RS-13) (27). Ambivalence regarding living donation was assessed using the Simmons scale (28).

To identify donors with clinically relevant impairment, a cut-off value of 10 was used for PHQ-9, GAD-7, and PHQ-15. For SF-12 and MFI-20, a value of more than one standard deviation from the mean of the age- and gender-adjusted German norm was defined as a clinically relevant impairment.

### Statistics

Categorical variables were presented as absolute and relative frequencies and compared between two groups using Fisher's exact test. Continuous variables were presented as mean ± standard deviation, median, and 25 and 75% quantiles. Depending on the distribution, *t*-tests or Mann–Whitney *U* tests were used to compare continuous variables.

Logistic regression was performed for renal function (cut-off 80 ml/min/1.73 m^2^), and linear regressions were performed for quality of life and fatigue scales.

The following variables were defined as cardiovascular risk factors: active smoking status, medication for diabetes, hypertension, lipid-lowering drugs, HbA1c value >6.5%, BMI > 30, and previous heart, blood, or blood vessel disease.

No imputation for missing data was performed.

*P*-values < 0.05 were considered statistically significant. Statistical analysis was performed using SAS 9.4 (SAS Institute, Cary, NC, USA).

## Results

### Donor recruitment

In the study period, centers reported *N* = 1,898 living donations. As they were not sufficiently fluent in German, Turkish, or Russian, 9% (*n* = 173) did not meet the language inclusion criteria. The remaining 91% (*n* = 1,725) constituted the group of recruitable donors. Of these, 18.6% (*n* = 321) were not recruited for the following reasons: 10% (5.6%−14.5% yearly) did not give their written consent to participate in the survey, and 8.6% were not recruited for organizational reasons, for example, staff shortages. Because at the time of data export it was not yet known whether the living donation surgery had been performed and whether these individuals were in fact living donors, 9.7% (*n* = 167) had to be excluded for the analysis. Thus, 72% (*n* = 1,237) of recruitable donors were included in the analysis.

### Donor cohort

Pre-operative data from 1,237 donors (1/2020-1/2025), who subsequently underwent surgery, were evaluated. Donor questionnaires were completed at a median of 1 day (1st quartile: 1.00; 3rd quartile: 5.00) prior to donation. In total, 33.71% of living kidney donations were pre-emptive, 25.53% were AB0-incompatible, and 17.24% were immunological risk transplants defined by two requirements: (1) presence of donor-specific antibodies, and (2) desensitization (including plasma exchange or immunoadsorption). Regarding the surgical procedure, 59.47% of donors underwent laparoscopic nephrectomy, 20.79% underwent retroperitoneoscopic nephrectomy, and 19.74% underwent open nephrectomy.

### Sociodemographic data of donors

Average donor age was 54.48 ± 10.34 years (age range: 20–83 years); 62.81% were women, whose median age was 55 years; 27.54% of women were younger than 50 years and were, therefore, in the potentially reproductive phase of life. Donors had a relatively high level of education: 77.58% of women and 70.08% of men had a secondary school leaving certificate or higher. Regarding employment, 78.35% of donors were employed at the time of donation, with 41.03% of women working full-time compared to 72.71% of men (*P* < 0.001; Table 1).

**Table 1:** Demographic data of donors.

Variable	Value	Total	Women	Men	*P*-value
Gender (*N* = 1,237)		1,237	777 (62.81%)	460 (37.19%)	
Age (*N* = 1,237)^◇^		1,237; 54.48 ± 10.34; 55.0 (48.00; 61.00)	777; 54.55 ± 10.06; 55.00 (49.00; 61.00)	460; 54.36 ± 10.82; 56.00 (48.00; 62.00)	0.758
Ethnicity (*N* = 1,221)	European (white skin color)	1,174 (96.15%)	734 (95.95%)	440 (96.49%)	0.406
	African/African American (black skin color)	5 (0.41%)	≤ 3 (0.39%)	≤ 3 (0.44%)	
	South American (Hispanic)	≤ 3 (0.16%)	≤ 3 (0.26%)	0	
	Asian (even if born in Europe)	21 (1.72%)	11 (1.44%)	10 (2.19%)	
	Other	19 (1.56%)	15 (1.96%)	4 (0.88%)	
Born in Germany	Yes	1,076 (87.06%)	676 (87.00%)	400 (87.15%)	1.000
Highest school certificate (*N* = 1,234)	No school certificate	22 (1.78%)	17 (2.19%)	5 (1.09%)	< 0.001
	Elementary school/secondary school/polytechnic secondary school	265 (21.47%)	143 (18.43%)	122 (26.64%)	
	Secondary school leaving certificate (intermediate school leaving certificate)/polytechnic secondary school	494 (40.03%)	351 (45.23%)	143 (31.22%)	
	Technical college entrance qualification (technical secondary school leaving certificate)/general higher education entrance qualification (A-levels)	429 (34.76%)	251 (32.35%)	178 (38.86%)	
	Other school leaving certificate	24 (1.94%)	14 (1.80%)	10 (2.18%)	
Vocational qualification (*N* = 1,231)	No vocational qualification	99 (8.04%)	65 (8.41%)	34 (7.42%)	< 0.001
	Apprenticeship/vocational school qualification	726 (58.98%)	506 (65.46%)	220 (48.03%)	
	Technician/master craftsman school	109 (8.85%)	31 (4.01%)	78 (17.03%)	
	Technical college/university degree	297 (24.13%)	171 (22.12%)	126 (27.51%)	
Employment (*N* = 1,233)	Yes. full-time	651 (52.80%)	318 (41.03%)	333 (72.71%)	< 0.001
	Yes, part-time regularly at least 15 h/week	253 (20.52%)	229 (29.55%)	24 (5.24%)	
	Yes, part-time irregularly or less than 15 h/week	62 (5.03%)	53 (6.84%)	9 (1.97%)	
	No, not employed	267 (21.65%)	175 (22.58%)	92 (20.09%)	
Marital/relationship status (*N* = 1,233)	Committed relationship and living with partner	1,037 (84.10%)	651 (84.00%)	386 (84.28%)	< 0.001
	Committed relationship but living apart from partner	42 (3.41%)	23 (2.97%)	19 (4.15%)	
	Single	52 (4.22%)	22 (2.84%)	30 (6.55%)	
	Divorced	71 (5.76%)	51 (6.58%)	20 (4.37%)	
	Widowed	31 (2.51%)	28 (3.61%)	3 (0.66%)	
Time off sick in the last 12 months [weeks] (*N* = 254)		254; 5.45 ± 8.48; 3.00 (2.00; 6.00)	168; 5.57 ± 8.54; 3.00 (2.00; 6.00)	86; 5.21 ± 8.40; 3.00 (2.00; 5.00)	0.370
Children (*N* = 1,231)	Yes	1,004 (81.56%)	659 (85.03%)	345 (75.66%)	< 0.001
Number of children (*N* = 984)		984; 2.12 ± 0.90; 2.00 (2.00; 2.00)	649; 2.12 ± 0.88; 2.00 (2.00; 2.00)	335; 2.12 ± 0.94; 2.00 (2.00; 3.00)	0.868
Care-dependent persons in one's household (except recipient; *N* = 1,231)	Yes, children under age 18	129 (10.48%)	84 (10.85%)	45 (9.85%)	0.238
	Yes, relatives requiring care	46 (3.74%)	34 (4.39%)	12 (2.63%)	
	Yes, children under 18 and relatives in need of care	≤ 3 (0.24%)	≤ 3 (0.13%)	≤ 3 (0.44%)	
	No	1,053 (85.54%)	655 (84.63%)	398 (87.09%)	
General health perception (*N* = 1,209)	Excellent	177 (14.64%)	102 (13.33%)	75 (16.89%)	0.352
	Very good	709 (58.64%)	458 (59.87%)	251 (56.53%)	
	Good	320 (26.47%)	203 (26.54%)	117 (26.35%)	
	Less good	≤ 3 (0.25%)	≤ 3 (0.26%)	≤ 3 (0.23%)	

Diamond ◇ = normal distribution; representation: N = number (percentage); mean ± standard deviation; median (1st quartile; 3rd quartile).

**Figure 1:**
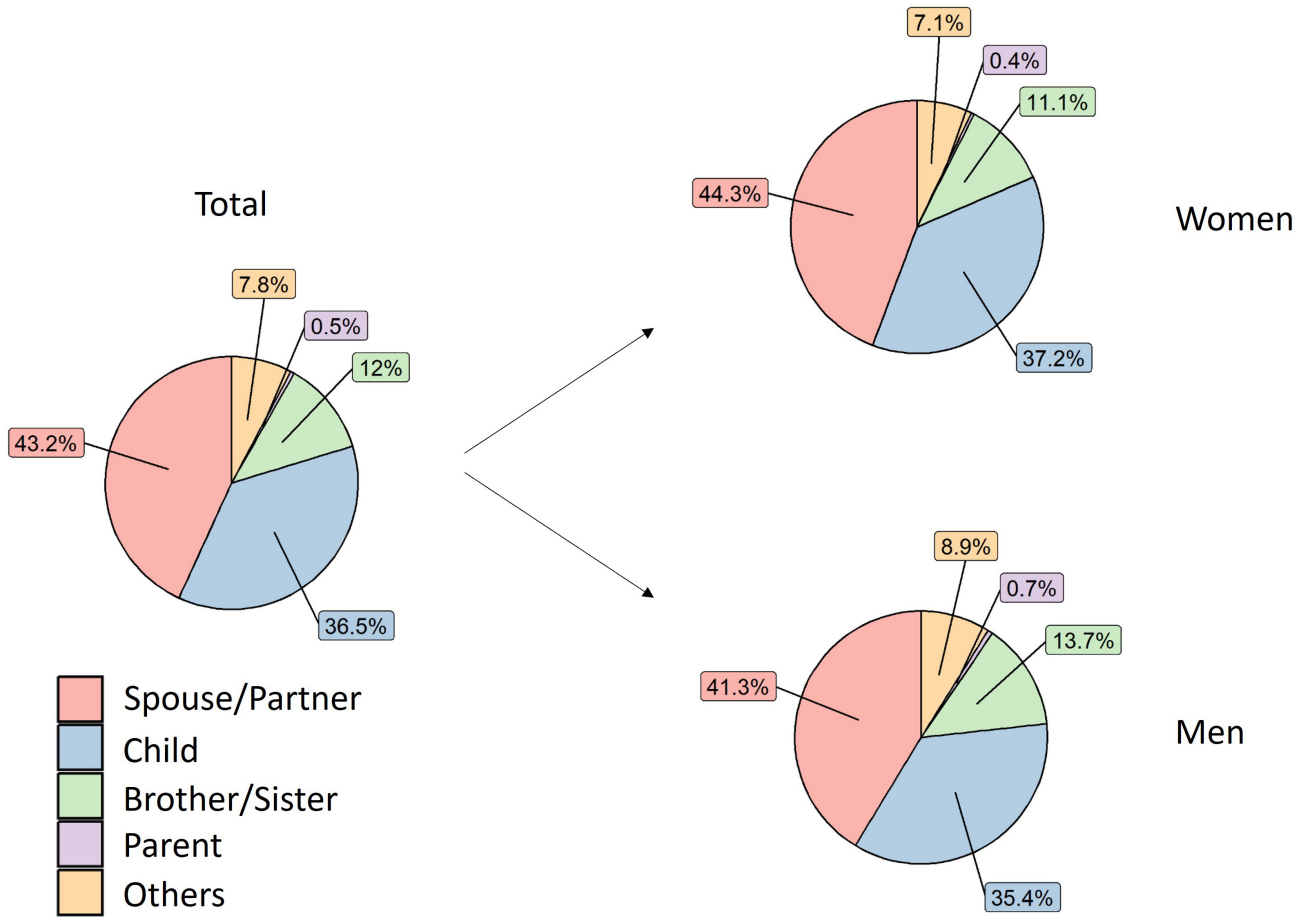
Who donates to whom?

Both women and men most frequently donated to their children or partners (Table 2, Figure 1). Of the women, 85.03% had children of their own, compared to 75.66% of men (*P* < 0.001). There was no difference between men and women in terms of the number of people they had to care for in their household (Table 1). Almost half of the donors (45.71%; Table 2) were involved in caring for or supporting the organ recipient (women 47.29% vs. men 43.02%; *P* = 0.168). Of these, 15.91% felt “severely” or “very severely” burdened by caring for/supporting the organ recipient (women 18.77% vs. men 10.53%; *P* = 0.014).

**Table 2:** Donation initiative and factors.

Variable	Value	Total	Women	Men	*P*-value
For whom did you donate? (*N* = 1,237)	Spouse/partner	534 (43.17%)	344 (44.27%)	190 (41.30%)	0.363
	Child	452 (36.54%)	289 (37.19%)	163 (35.43%)	
	Brother/sister	149 (12.05%)	86 (11.07%)	63 (13.70%)	
	Parent	6 (0.49%)	3 (0.39%)	3 (0.65%)	
	Other	96 (7.76%)	55 (7.08%)	41 (8.91%)	
Initiative to donate came from... (*N* = 1,231)	Donor themselves	1,133 (92.04%)	727 (94.17%)	406 (88.45%)	< 0.001
	A member of the donor's family	8 (0.65%)	≤ 3 (0.13%)	7 (1.53%)	
	Recipient	48 (3.90%)	21 (2.72%)	27 (5.88%)	
	Relatives of the recipient	≤ 3 (0.08%)	–	≤ 3 (0.22%)	
	Medical staff	40 (3.25%)	22 (2.85%)	18 (3.92%)	
	Others	≤ 3 (0.08%)	≤ 3 (0.13%)	–	
Time to make the decision (*N* = 1,230)	< 3 months	1,087 (88.37%)	679 (88.07%)	408 (88.89%)	0.372
	3–6 months	73 (5.93%)	50 (6.49%)	23 (5.01%)	
	6–12 months	33 (2.68%)	17 (2.20%)	16 (3.49%)	
	>12 months	37 (3.01%)	25 (3.24%)	12 (2.61%)	
Duration of medical diagnostics and preparation for donation (*N* = 1,231)	< 3 months	77 (6.26%)	42 (5.44%)	35 (7.63%)	0.338
	3–6 months	284 (23.07%)	177 (22.93%)	107 (23.31%)	
	6–12 months	473 (38.42%)	294 (38.08%)	179 (39.00%)	
	>12 months	397 (32.25%)	259 (33.55%)	138 (30.07%)	
Donor's assessment of the duration of preparation for donation (*N* = 1,225)	Too long	565 (46.12%)	360 (46.81%)	205 (44.96%)	0.378
	Too short	3 (0.24%)	3 (0.39%)	–	
	Just right	657 (53.63%)	406 (52.80%)	251 (55.04%)	
Information provided by the transplant center about living donation (*N* = 1,235)	Very good	820 (66.40%)	515 (66.28%)	305 (66.59%)	0.721
	Good	376 (30.45%)	237 (30.50%)	139 (30.35%)	
	Moderate	29 (2.35%)	20 (2.57%)	9 (1.97%)	
	Poor	≤ 3 (0.08%)	–	≤ 3 (0.22%)	
	Very poor	9 (0.73%)	5 (0.64%)	4 (0.87%)	
Felt pressured to donate (*N* = 1,234)	Not at all	1,210 (98.06%)	758 (97.68%)	452 (98.69%)	0.030
	Somewhat	20 (1.62%)	17 (2.19%)	3 (0.66%)	
	Moderate	≤ 3 (0.08%)	–	≤ 3 (0.22%)	
	Strong	≤ 3 (0.16%)	≤ 3 (0.13%)	≤ 3 (0.22%)	
	Very strong	≤ 3 (0.08%)	–	≤ 3 (0.22%)	
Involvement in the care/treatment of the recipient prior to living donation (*N* = 1,201)	Yes	549 (45.71%)	358 (47.29%)	191 (43.02%)	0.168
Stress caused by involvement in the care/treatment of the recipient prior to living donation (*N* = 547)	Not at all	208 (38.03%)	134 (37.54%)	74 (38.95%)	0.063
	Somewhat	132 (24.13%)	84 (23.53%)	48 (25.26%)	
	Moderate	120 (21.94%)	72 (20.17%)	48 (25.26%)	
	Severe	59 (10.79%)	48 (13.45%)	11 (5.79%)	
	Very severe	28 (5.12%)	19 (5.32%)	9 (4.74%)	
	Not at all/Somewhat/Moderate	460 (84.10%)	290 (81.23%)	170 (89.47%)	0.014
	Severe/Very severe	87 (15.90%)	67 (18.77%)	20 (10.53%)	
Did you... (*N* = 1,234)	I knew immediately that I would definitely donate	1,032 (83.63%)	665 (85.70%)	367 (80.13%)	0.013
	I had to think about it first	202 (16.37%)	111 (14.30%)	91 (19.87%)	
How difficult was it for you to decide whether to donate? Would you say the decision was... (*N* = 1,234)	Very difficult	≤ 3 (0.16%)	≤ 3 (0.13%)	≤ 3 (0.22%)	0.003
	Moderately difficult	9 (0.73%)	6 (0.77%)	3 (0.65%)	
	A little difficult	79 (6.40%)	42 (5.42%)	37 (8.06%)	
	Not difficult at all	669 (54.21%)	398 (51.35%)	271 (59.04%)	
	It wasn't a decision at all. I offered my donation spontaneously.	475 (38.49%)	328 (42.32%)	147 (32.03%)	

Representation: N = number (percentage); mean ± standard deviation; median (1st quartile; 3rd quartile).

### Donor initiative and associated factors

Most donors stated that the initiative to donate came from themselves (women 94.17% vs. men 88.45%; *P* < 0.001), and 85.70% of women and 80.13% of men (*P* = 0.013) stated that they “knew immediately that they would donate” (Table 2). When asked “How difficult was it for you to decide whether to donate?,” 42.32% of women and 32.03% of men (*P* = 0.003) responded that they had offered to donate spontaneously. Men were more often encouraged to donate by their family, the organ recipient, or the organ recipient's family (women 2.85% vs. men 7.63%; *P* < 0.001). Rarely did the initiative to donate come from the medical side (women 2.85% vs. men 3.92%; *P* < 0.001).

In total, 2.32% of women and 1.32% of men (*P* = 0.030) felt pressured to donate, most of them felt “somewhat” pressured. Of all 1,234 donors, just four (0.32%) rated feeling “moderate” to “very strong” pressure to donate; all four donated to their child.

Both men and women made the decision to donate quickly (88.37% ≤ 3 months). For 70.67%, the medical preparation took more than 6 months; 53.63% were satisfied, while 46.12% felt the preparation was “too long.”

Women and men rated the information and education provided by the transplant center as “very good” or “good” (96.85%). Few (0.81%) rated the education process as “poor” or “very poor.”

### Health status of living kidney donors

In total, 99.75% described their general health as “good” to “excellent” (Table 1), although only 44.08% reported no pre-existing conditions or regular medication (women 40.81% vs. men 49.59%; *P* = 0.008; Tables 3A, B). Hypertension treated with antihypertensive medication was reported by 27.27% of donors prior to donation.

**Table 3A:** Pre-existing conditions of living kidney donors.

Variable	Value	Total	Women	Men	*P*-value Women vs. Men
Pre-existing conditions					
Pre-existing conditions affecting the heart, blood or blood vessels (*N* = 1,233)	Yes	62 (5.03%)	35 (4.53%)	27 (5.87%)	0.346
Heart attack or stents in the heart (*N* = 53)	Yes	≤ 3 (1.89%)	0	≤ 3 (4.17%)	0.453
Circulatory disorders or stents in the leg vessels (intermittent claudication; *N* = 54)	Yes	4 (7.41%)	≤ 3 (3.45%)	≤ 3 (12.00%)	0.326
Stroke (*N* = 54)	Yes	≤ 3 (1.85%)	0	≤ 3 (4.00%)	0.463
Thrombosis (*N* = 53)	Yes	9 (16.98%)	6 (21.43%)	3 (12.00%)	0.474
Coagulation disorders (*N* = 52)	Yes	≤ 3 (5.77%)	≤ 3 (6.90%)	≤ 3 (4.35%)	1.000
Other (*N* = 58)	Yes	34 (58.62%)	18 (51.43%)	16 (69.57%)	0.188
Malignant pre-existing conditions (tumor; *N* = 1,230)	Yes	48 (3.90%)	29 (3.76%)	19 (4.15%)	0.762
Colorectal cancer (*N* = 28)	Yes	4 (14.29%)	≤ 3 (6.67%)	≤ 3 (23.08%)	0.311
Breast cancer (*N* = 31)	Yes	6 (19.35%)	6 (33.33%)	0	0.028
Prostate cancer (*N* = 14)	Yes	7 (50.00%)	-	7 (50.00%)	
Malignant melanoma (*N* = 29)	Yes	4 (13.79%)	≤ 3 (18.75%)	≤ 3 (7.69%)	0.606
Non-melanoma skin cancer/basal cell carcinoma (*N* = 33)	Yes	10 (30.30%)	5 (27.78%)	5 (33.33%)	1.000
Kidney cancer (*N* = 28)	Yes	0 (100.00%)	0	0	
Other (*N* = 36)	Yes	18 (50.00%)	14 (60.87%)	4 (30.77%)	0.164
Immune system diseases/autoimmune diseases (*N* = 1,223)	Yes	67 (5.48%)	56 (7.31%)	11 (2.41%)	< 0.001
Hashimoto's disease (thyroid; *N* = 51)	Yes	33 (64.71%)	31 (72.09%)	≤ 3 (25.00%)	0.017
Chronic inflammatory bowel disease (Crohn's disease or ulcerative colitis; *N* = 42)	Yes	≤ 3 (4.76%)	≤ 3 (5.71%)	0	1.000
Psoriasis (*N* = 42)	Yes	12 (28.57%)	7 (20.59%)	5 (62.50%)	0.031
Rheumatic disorders (rheumatism, rheumatoid arthritis, joint rheumatism; *N* = 43)	Yes	10 (23.26%)	8 (22.22%)	≤ 3 (28.57%)	0.656
Other (*N* = 48)	Yes	16 (33.33%)	13 (33.33%)	≤ 3 (33.33%)	1.000
Chronic pain disorder (*N* = 1,224)	Yes	47 (3.84%)	37 (4.81%)	10 (2.20%)	0.021
Do you suffer from chronic fatigue (fatigue syndrome; *N* = 1,225)	Yes	≤ 3 (0.24%)	≤ 3 (0.26%)	≤ 3 (0.22%)	1.000
Previous mental health disorders (*N* = 1,218)	Yes	66 (5.42%)	47 (6.14%)	19 (4.19%)	0.153
Depression (*N* = 60)	Yes	48 (80.00%)	36 (83.72%)	12 (70.59%)	0.293
Anxiety disorders (n = 51)	Yes	16 (31.37%)	11 (31.43%)	5 (31.25%)	1.000
Obsessive-compulsive disorders (*N* = 46)	Yes	0 (100.00%)	0	0	
Addictions (*N* = 46)	Yes	≤ 3 (6.52%)	0	≤ 3 (20.00%)	0.030
Eating disorders (*N* = 47)	Yes	≤ 3 (6.38%)	≤ 3 (9.38%)	0	0.541
Psychoses (*N* = 47)	Yes	≤ 3 (6.38%)	≤ 3 (3.23%)	≤ 3 (12.50%)	0.264
Other (*N* = 45)	Yes	12 (26.67%)	10 (31.25%)	≤ 3 (15.38%)	0.460
Psychotherapeutic treatment (*N* = 1,207)	Yes	43 (3.56%)	36 (4.72%)	7 (1.57%)	0.004
Smoking status	I am a smoker	152 (12.31%)	91 (11.73%)	61 (13.29%)	0.004
	I used to smoke	553 (44.78%)	324 (41.75%)	229 (49.89%)	
	I have never smoked	530 (42.91%)	361 (46.52%)	169 (36.82%)	
Donor's information: high blood pressure (*N* = 1,235)	Yes	349 (28.26%)	210 (27.10%)	139 (30.22%)	0.240

Representation: N = number (percentage); Indented variables refer to the preceding variable; mean ± standard deviation; median (1st quartile; 3rd quartile).

**Table 3B:** Pre-existing medication of living kidney donors.

Variable	Value	Total	Women	Men	*P*-value Women vs. Men
Medication					
Doctor's information: Hypertension treated with medication (*N* = 1,232)	Yes	336 (27.27%)	195 (25.16%)	141 (30.85%)	0.034
Treated with…					
Beta blockers (*N* = 334)	Yes	63 (18.86%)	47 (24.23%)	16 (11.43%)	0.003
ACE inhibitors or angiotensin II blocker (*N* = 336)	Yes	276 (82.14%)	152 (77.95%)	124 (87.94%)	0.021
Calcium antagonists (*N* = 336)	Yes	120 (35.71%)	65 (33.33%)	55 (39.01%)	0.301
Diuretics (*N* = 336)	Yes	40 (11.90%)	26 (13.33%)	14 (9.93%)	0.396
Another anithypertensive medication (*N* = 325)	Yes	9 (2.77%)	6 (3.19%)	3 (2.19%)	0.739
>2 antihypertensive drugs (doctor's prescription; *N* = 336)	Yes	29 (8.71%)	20 (10.36%)	9 (6.43%)	0.241
Donor's information: diabetes (high blood sugar)... (*N* = 1,229)	Yes	5 (0.41%)	≤ 3 (0.39%)	≤ 3 (0.44%)	1.000
Tablets (*N* = 5)	Yes	5 (100.00%)	≤ 3 (100.00%)	≤ 3 (100.00%)	
Insulin injections (*N* = 4)	Yes	0 (100.00%)			
15.6-7.5,-14.1499ptDoctor's information: diabetes mellitus is treated with medication? (*N* = 1,230)	Yes	4 (0.33%)	≤ 3 (0.26%)	≤ 3 (0.44%)	0.630
Treated with…					
Tablets (*N* = 4)	Yes	4 (100.00%)	≤ 3 (100.00%)	≤ 3 (100.00%)	
Insulin injections (*N* = 4)	Yes	0 (100.00%)			
Donor's information: blood thinners (*N* = 1,219)	Yes	33 (2.71%)	12 (1.56%)	21 (4.65%)	0.003
Donor's information: blood lipid-lowering drugs (*N* = 1,217)	Yes	109 (8.96%)	56 (7.29%)	53 (11.80%)	0.009
Donor‘s information: painkillers (*N* = 1,220)	Yes	57 (4.67%)	46 (5.99%)	11 (2.43%)	0.005
Donor's information: sleeping pills (*N* = 1,208)	Yes	53 (4.39%)	44 (5.77%)	9 (2.02%)	0.002
Donor‘s information: antidepressants (*N* = 1,217)	Yes	24 (1.97%)	21 (2.75%)	3 (0.66%)	0.010
Donor's information: psychiatric medication (including antidepressants; *N* = 1,217)	Yes	29 (2.38%)	23 (3.01%)	6 (1.32%)	0.079
Donor‘s information: medication to treat restlessness (*N* = 1,215)	Yes	30 (2.47%)	23 (2.99%)	7 (1.57%)	0.178
Donor's information: other medications (*N* = 1,105)	Yes	208 (18.82%)	152 (22.06%)	56 (13.46%)	< 0.001
At least one pre-existing condition or medication (donor's information; *N* = 989)	Yes	553 (55.92%)	367 (59.19%)	186 (50.41%)	0.008
At least one cardiovascular risk factor	Yes	572 (50.75%)	349 (49.29%)	223 (53.22%)	0.218
Number of cardiovascular risk factors		1,127 0.68 ± 0.78; 1.00 (0.00; 1.00)	708 0.64 ± 0.75; 0.00 (0.00; 1.00)	419 0.75 ± 0.83; 1.00 (0.00; 1.00)	0.049

Representation: N = number (percentage); Indented variables refer to the preceding variable; mean ± standard deviation; median (1st quartile; 3rd quartile); information on dietary supplements and vitamin substitution was not taken into account.

The mean BMI (26.06 ± 3.54 kg/m^2^) indicated slight overweight; 13.9% were obese, including 0.89% with grade II obesity (Table 4). More men were among the 12.31% who were active smokers (*P* = 0.004); under half had never smoked (women 46.52% vs. men 36.82%, *P* = 0.004; Table 3A).

**Table 4:** Clinical data of donors.

Variable	Characteristic	Total	Women	Men	*P*-value
Creatinine [mg/dl] (*N* = 1,163)^◇^		1,163; 0.81 ± 0.14; 0.80 (0.70; 0.90)	731; 0.74 ± 0.11; 0.74 (0.68; 0.80)	432; 0.93 ± 0.12; 0.92 (0.86; 1.00)	< 0.001
eGFR CKD-EPI_2021 (*N* = 1,163)^◇^		1,163; 94.69 ± 12.78; 95.49 (85.67; 103.33)	731; 94.25 ± 13.05; 95.41 (84.76; 103.79)	432; 95.45 ± 12.28; 95.86 (87.21; 102.86)	0.122
eGFR CKD-EPI_2021 (*N* = 1,163)	>90	738 (63.46%)	450 (61.56%)	288 (66.67%)	0.089
	60– ≤ 90	425 (36.54%)	281 (38.44%)	144 (33.33%)	
	< 60	0	0	0	
eGFR CKD-EPI_2021 (*N* = 1,163)	>80	1,006 (86.50%)	623 (85.23%)	383 (88.66%)	0.110
	≤ 80	157 (13.50%)	108 (14.77%)	49 (11.34%)	
Hemoglobin [g/dl] (*N* = 1,165)^◇^		1,165; 14.14 ± 1.16; 14.02 (13.40; 14.90)	731; 13.61 ± 0.89; 13.60 (13.10; 14.20)	434; 15.02 ± 1.02; 15.00 (14.30; 15.70)	< 0.001
HbA1c [%] (*N* = 1,135)^◇^		1,135; 5.44 ± 0.30; 5.40 (5.20; 5.60)	711; 5.43 ± 0.31; 5.40 (5.20; 5.60)	424; 5.44 ± 0.30; 5.40 (5.20; 5.70)	0.602
Serum/plasma glucose [mg/dl] (fasting blood sample; *N* = 318)		318; 94.27 ± 12.22; 94.00 (87.00; 100.00)	199; 94.20 ± 12.49; 93.16 (86.00; 100.00)	119; 94.37 ± 11.82; 94.00 (87.00; 101.00)	0.604
Cholesterol [mg/dl] (*N* = 1,145)		1,145; 210.69 ± 40.52; 209.00 (183.00; 237.00)	716; 214.29 ± 41.11; 212.69 (185.00; 241.00)	429; 204.67 ± 38.82; 204.00 (180.00; 229.00)	< 0.001
LDL cholesterol [mg/dl] (*N* = 1,070)^◇^		1,070; 131.64 ± 35.92; 130.50 (106.00; 155.00)	672; 131.18 ± 36.33; 129.00 (105.00; 156.00)	398; 132.43 ± 35.24; 132.50 (108.00; 154.29)	0.581
HDL cholesterol [mg/dl] (*N* = 1,068)^◇^		1,068; 62.70 ± 17.14; 61.00 (50.00; 73.00)	671; 68.37 ± 16.67; 66.13 (56.46; 78.00)	397; 53.11 ± 13.21; 51.00 (44.00; 61.00)	< 0.001
TSH [mU/L] (*N* = 1,157)		1,157; 1.74 ± 1.05; 1.52 (1.07; 2.19)	731; 1.76 ± 1.09; 1.54 (1.02; 2.28)	426; 1.71 ± 0.97; 1.51 (1.10; 2.06)	0.612
Albumin inspontaneous or 24 h collected urine [mg/L] (*N* = 1,011)		1,011; 5.27 ± 9.16; 3.00 (0.00; 7.10)	645; 4.88 ± 8.77; 2.00 (0.00; 6.80)	366; 5.94 ± 9.79; 3.00 (0.00; 8.00)	0.053
Albumin/creatinine ratio in spontaneous or 24h collected urine [mg/g] (*N* = 893)		893; 6.34 ± 15.91; 2.16 (0.00; 7.10)	561; 6.23 ± 10.94; 0.00 (0.00; 8.00)	332; 6.52 ± 21.89; 2.74 (0.00; 6.40)	0.802
Microhaematuria (*N* = 1,042)	Yes	81 (7.77%)	67 (10.29%)	14 (3.58%)	< 0.001
BMI (*N* = 1,237)^◇^		1,237; 26.06 ± 3.54; 25.95 (23.51; 28.38)	777; 25.73 ± 3.71; 25.51 (23.01; 28.09)	460; 26.62 ± 3.14; 26.59 (24.34; 28.67)	< 0.001
Weight classification according to the Obesity Society (*N* = 1,237)	Underweight	4 (0.32%)	4 (0.51%)		< 0.001
	Normal weight	504 (40.74%)	354 (45.56%)	150 (32.61%)	
	Overweight	557 (45.03%)	314 (40.41%)	243 (52.83%)	
	Obesity grade I	161 (13.02%)	96 (12.36%)	65 (14.13%)	
	Obesity grade II	≤ 12 (0.89%)	9 (1.16%)	≤ 3 (0.43%)	
Systolic blood pressure, clinic (*N* = 977)^◇^		977; 131.89 ± 15.55; 130.00 (120.00; 140.00)	624; 130.60 ± 16.81; 130.00 (120.00; 140.00)	353; 134.16 ± 12.76; 133.00 (125.00; 142.00)	< 0.001
Diastolic blood pressure. clinic (*N* = 977)^◇^		977; 80.81 ± 10.03; 80.00 (75.00; 87.00)	624; 80.08 ± 10.45; 80.00 (73.00; 86.00)	353; 82.08 ± 9.12; 80.00 (77.00; 88.00)	0.002
Mean arterial blood pressure (MAD; *N* = 977)^◇^		977; 97.83 ± 10.69; 97.00 (91.00; 104.33)	624; 96.92 ± 11.36; 96.67 (89.33; 103.33)	353; 99.44 ± 9.18; 98.33 (93.33; 105.33)	< 0.001
24-h systolic blood pressure (*N* = 782)^◇^		782; 124.44 ± 11.25; 124.00 (117.00; 131.00)	474; 123.36 ± 11.69; 123.00 (116.00; 130.00)	308; 126.11 ± 10.33; 126.00 (119.00; 132.50)	< 0.001
24-h diastolic blood pressure (*N* = 782)^◇^		782; 77.37 ± 7.82; 77.00 (72.00; 82.00)	474; 76.42 ± 7.98; 76.00 (71.00; 81.00)	308; 78.83 ± 7.34; 79.00 (74.00; 83.00)	< 0.001
Day-night rhythm present (dipping >20%; *N* = 740)	Yes	192 (25.95%)	115 (25.84%)	77 (26.10%)	0.932

Diamond ◇ = normal distribution; representation: N = number (percentage); mean ± standard deviation; median (1st quartile; 3rd quartile).

Regarding cardiovascular risk factors, 50.75% had one or more risk factors (no gender difference), but men had a higher number of risk factors (*P* = 0.049). Only 3.90% had a history of malignancy. Women were more likely to have autoimmune diseases (women 7.31% vs. men 2.41%; *P* < 0.001) and chronic pain disorders (women 4.81% vs. men 2.20%; *P* = 0.021). Overall, 0.24% reported suffering from chronic fatigue syndrome, and 5.42% reported previous mental health disorders, primarily depressive and anxiety disorders. No gender difference was found in previous mental health disorders, but more women were in psychotherapeutic treatment (women 4.72% vs. men 1.57%; *P* = 0.004) and were receiving antidepressant medication (women 2.75% vs. men 0.66%; *P* = 0.010).

The eGFR-CKD-EPI (2021) was within the normal range (94.69 ± 12.78 ml/min/1.73m^2^; range: 60.99–130.61 ml/min/1.73 m^2^). In 13.50%, renal function was below 80 ml/min/1.73 m^2^ at a mean age of 59.93 ± 7.85 years (age range: 39–83 years; women: 59.03 ± 8.36; men: 61.92 ± 6.21; *P* = 0.017). In the multivariate analysis, only age significantly influenced renal function (Supplementary Table S1, Figures S2F–H). In 3.70% of cases, an albumin/creatinine ratio >30 mg/g was found. Microhaematuria was observed in 7.77% of cases (women 10.29% vs. men 3.58%; *P* < 0.001). Microhaematuria was detected in 12.72% of women of childbearing age. Further clinical parameters are shown in Table 4.

### Standardized psychological questionnaires

**Figure 2:**
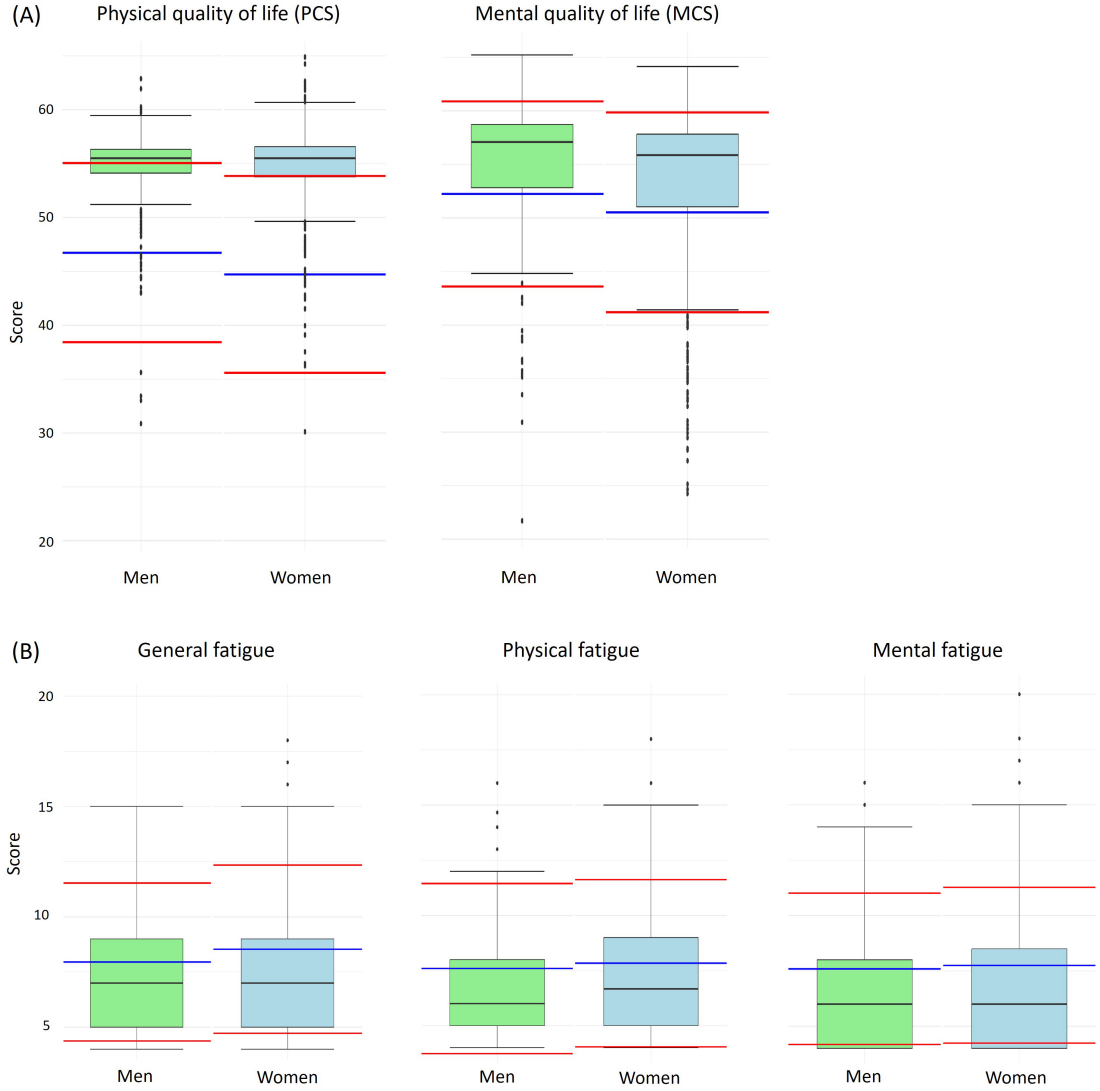
**(A)** Results of physical (left) and mental (right) quality of life from the SF-12 self-report questionnaire before living kidney donation for women (blue) and men (green). Data were compared with mean values for the 51- to 60-year-old standard sample (46.74 (PCS) and 52.24 (MCS) for men and 44.72 (PCS) and 50.52 (MCS) for women, blue line) ± standard deviation (8.33 (PCS) and 8.65 (MCS) for men and 9.13 (PCS) and 9.34 (MCS) for women, red line). **(B)** Results of general (left), physical (middle), and mental (right) fatigue from the MFI-20 self-report questionnaire before living kidney donation for women (blue) and men (green). Data were compared with mean values for the 45- to 54-year-old standard sample, 7.95 (general fatigue), 7.75 (physical fatigue) and 7.59 (mental fatigue) for men and 8.53 (general fatigue), 7.83 (physical fatigue) and 7.75 (mental fatigue) for women, blue line) ± standard deviation, 3.58 (general fatigue), 3.86 (physical fatigue) and 3.42 (mental fatigue) for men and 3.82 (general fatigue), 3.79 (physical fatigue) and 3.53 (mental fatigue) for women, red line).

Table 5 shows the results of the standardized psychological questionnaires. While no difference was found between the sexes for physical quality of life (*P* = 0.939), women had a lower mean score for mental quality of life (*P* < 0.001). Women also had lower mean scores on the psychological symptom scales (depression, anxiety, and somatic symptom severity) and the scales for general fatigue and reduced motivation. However, both genders showed significantly better values than the respective age-adjusted norm in all domains (*P* < 0.001; Table 6, Figures 2A, B).

**Table 5:** Results of the standardized psychological questionnaires.

Variable	Total	Women	Men	*P*-value Women vs. Men
SF-12 Physical health summary score (quality of life; *N* = 1,175)	1,175; 54.71 ± 3.41; 55.50 (53.80; 56.49)	739; 54.72 ± 3.42; 55.50 (53.80; 56.58)	436; 54.70 ± 3.40; 55.50 (54.13; 56.34)	0.939
SF-12 Physical health summary score >1 SD below mean (*N* = 1,175)^*^	6 (0.51%)	≤ 3 (0.27%)	4 (0.91%)	0.202
SF-12 Mental health summary score (quality of life; *N* = 1,175)	1,175; 54.35 ± 6.24; 55.93 (52.04; 57.89)	739; 53.69 ± 6.70; 55.87 (51.02; 57.83)	436; 55.48 ± 5.19; 57.10 (52.82; 58.74)	< 0.001
SF-12 Mental health summary score >1 SD below mean (*N* = 1,175)^*^	66 (5.62%)	51 (6.90%)	15 (3.44%)	0.013
MFI-20 General fatigue (*N* = 1,195)^◇^	1,195; 7.27 ± 2.82; 7.00 (5.00; 9.00)	757; 7.45 ± 2.95; 7.00 (5.00; 9.00)	438; 6.97 ± 2.55; 7.00 (5.00; 9.00)	0.004
MFI-20 General fatigue >1 SD above mean (*N* = 1,195)^*^	58 (4.85%)	34 (4.49%)	24 (5.48%)	0.486
MFI-20 Physical fatigue (*N* = 1,193)^◇^	1,193; 7.03 ± 2.66; 6.00 (5.00; 8.00)	755; 7.15 ± 2.72; 6.67 (5.00; 9.00)	438; 6.83 ± 2.54; 6.00 (5.00; 8.00)	0.046
MFI-20 Physical fatigue >1 SD above mean (*N* = 1,193)^*^	56 (4.69%)	37 (4.90%)	19 (4.34%)	0.777
MFI-20 Mental fatigue (*N* = 1,193)	1,193; 6.71 ± 2.88; 6.00 (4.00; 8.00)	755; 6.87 ± 3.02; 6.00 (4.00; 9.00)	438; 6.45 ± 2.58; 6.00 (4.00; 8.00)	0.084
MFI-20 Mental fatigue >1 SD above mean (*N* = 1,193)^*^	86 (7.21%)	65 (8.61%)	21 (4.79%)	0.015
MFI-20 Reduced activity (*N* = 1,193)^◇^	1,193; 7.09 ± 2.60; 7.00 (5.00; 9.00)	755; 7.09 ± 2.67; 7.00 (5.00; 9.00)	438; 7.09 ± 2.48; 7.00 (5.00; 9.00)	0.991
MFI-20 Reduced activity >1 SD above mean (*N* = 1,193)^*^	57 (4.78%)	38 (5.03%)	19 (4.34%)	0.673
MFI-20 Reduced motivation (*N* = 1,193)^◇^	1,193; 7.14 ± 2.55; 7.00 (5.00; 9.00)	756; 7.27 ± 2.67; 7.00 (5.00; 9.00)	437; 6.90 ± 2.31; 7.00 (5.00; 8.00)	0.012
MFI-20 Reduced motivation >1 SD above mean (*N* = 1,193)^*^	72 (6.04%)	47 (6.22%)	25 (5.72%)	0.801
PHQ-9 Depressive symptoms (*N* = 1,198)	1,198; 1.83 ± 2.09; 1.00 (0.00; 3.00)	758; 2.05 ± 2.20; 1.06 (0.00; 3.00)	440; 1.45 ± 1.83; 1.00 (0.00; 2.00)	< 0.001
PHQ-9 Depressive symptoms total score ≥10 (*N* = 1,198)	9 (0.75%)	8 (1.06%)	≤ 3 (0.23%)	0.167
PHQ-15 Somatic symptom severity (*N* = 1,197)	1,197; 2.50 ± 2.36; 2.00 (1.00; 4.00)	758; 2.76 ± 2.43; 2.00 (1.00; 4.00)	439; 2.05 ± 2.16; 1.07 (0.00; 3.21)	< 0.001
PHQ-15 Somatic symptom severity total score ≥10 (*N* = 1,197)	14 (1.17%)	12 (1.58%)	≤ 3 (0.46%)	0.097
GAD-7 Anxiety symptoms (*N* = 1,196)	1,196; 1.99 ± 2.32; 1.00 (0.00; 3.00)	757; 2.29 ± 2.48; 2.00 (0.00; 4.00)	439; 1.49 ± 1.92; 1.00 (0.00; 2.00)	< 0.001
GAD-7 Anxiety symptoms total score ≥10 (*N* = 1,196)	14 (1.17%)	12 (1.58%)	≤ 3 (0.46%)	0.097
PHQ Stress (*N* = 1,192)	1,192; 1.80 ± 2.00; 1.00 (0.00; 3.00)	753; 1.98 ± 2.11; 1.00 (0.00; 3.00)	439; 1.51 ± 1.76; 1.00 (0.00; 2.00)	< 0.001
RS-13 Resilience scale (*N* = 1,191)	1,191; 75.29 ± 17.82; 80.00 (73.00; 86.00)	754; 74.98 ± 18.33; 80.00 (73.00; 86.00)	437; 75.82 ± 16.90; 80.00 (74.00; 86.00)	0.967
Ambivalence scale (*N* = 1,164)	1,164; 1.43 ± 1.35; 1.00 (0.00; 2.00)	733; 1.36 ± 1.36; 1.00 (0.00; 2.00)	431; 1.56 ± 1.33; 1.00 (1.00; 2.00)	0.003

Diamond ◇ = normal distribution; representation: N = number (percentage); mean ± standard deviation; median (1st quartile; 3rd quartile).

^*^Number of donors with a score > 1 SD distance from the mean value of the age- and gender-adjusted German general population.

**Table 6:** Comparison of standardized psychological questionnaires with norm values for the general German population.

Variable	Output	Age- and gender-adjusted standard value	*P*-value *t*-test
SF-12 Physical health summary score Female (norm value: women aged 51–60)^◇^	739; 54.72 ± 3.42; 55.50 (53.80; 56.58)	44.72	< 0.001
SF-12 Physical health summary score Male (norm value: men aged 51–60)^◇^	436; 54.70 ± 3.40; 55.50 (54.13; 56.34)	46.74	< 0.001
SF-12 Mental health summary score Female (norm value: women aged 51–60)^◇^	739; 53.69 ± 6.70; 55.87 (51.02; 57.83)	50.53	< 0.001
SF-12 Mental health summary score Male (norm value: men aged 51–60)^◇^	436; 55.48 ± 5.19; 57.10 (52.82; 58.74)	52.24	< 0.001
MFI-20 General fatigue Female (norm value: women aged 45–54)^◇^	757; 7.45 ± 2.95; 7.00 (5.00; 9.00)	8.43	< 0.001
MFI-20 General fatigue Male (norm value: men aged 45–54)^◇^	438; 6.97 ± 2.55; 7.00 (5.00; 9.00)	7.95	< 0.001
MFI-20 Physical fatigue Female (norm value: women aged 45–54)^◇^	755; 7.15 ± 2.72; 6.67 (5.00; 9.00)	7.83	< 0.001
MFI-20 Physical fatigue Male (norm value: men aged 45–54)^◇^	438; 6.83 ± 2.54; 6.00 (5.00; 8.00)	7.59	< 0.001
MFI-20 Mental fatigue Female (norm value: women aged 45–54)^◇^	755; 6.87 ± 3.02; 6.00 (4.00; 9.00)	7.75	< 0.001
MFI-20 Mental fatigue Male (norm value: men aged 45–54)^◇^	438; 6.45 ± 2.58; 6.00 (4.00; 8.00)	7.59	< 0.001
MFI-20 Reduced activity Female (norm value: women aged 45–54)^◇^	755; 7.09 ± 2.67; 7.00 (5.00; 9.00)	7.56	< 0.001
MFI-20 Reduced activity Male (norm value: men aged 45–54)^◇^	438; 7.09 ± 2.48; 7.00 (5.00; 9.00)	7.75	< 0.001
MFI-20 Reduced motivation Female (normal value: women aged 45–54)^◇^	756; 7.27 ± 2.67; 7.00 (5.00; 9.00)	7.72	< 0.001
MFI-20 Reduced motivation Male (normal value: men aged 45–54)^◇^	437; 6.90 ± 2.31; 7.00 (5.00; 8.00)	7.74	< 0.001
PHQ-9 Depressive symptoms Female (normal value: women aged 45–54)^◇^	758; 2.05 ± 2.20; 1.06 (0.00; 3.00)	2.69	< 0.001
PHQ-9 Depressive symptoms Male (norm value: men aged 45–54)^◇^	440; 1.45 ± 1.83; 1.00 (0.00; 2.00)	2.86	< 0.001
PHQ-15 Somatic symptom severity Female (normal value: women aged 45–54)^◇^	758; 2.76 ± 2.43; 2.00 (1.00; 4.00)	3.80	< 0.001
PHQ-15 Somatic symptom severity Male (normal value: men aged 45–54)^◇^	439; 2.05 ± 2.16; 1.07 (0.00; 3.21)	3.30	< 0.001
GAD-7 Anxiety symptoms Female (normal value: women aged 45–54)^◇^	757; 2.29 ± 2.48; 2.00 (0.00; 4.00)	4.11	< 0.001
GAD-7 Anxiety symptoms Male (normal value: men aged 45–54)^◇^	439; 1.49 ± 1.92; 1.00 (0.00; 2.00)	3.00	< 0.001
PHQ Stress total sample (normal value: men + women aged 18–92)^◇^	1,192; 1.80 ± 2.00; 1.00 (0.00; 3.00)	2.54	< 0.001
RS-13 Resilience Female (normal value: women aged 45–54)^◇^	754; 74.98 ± 18.33; 80.00 (72.00; 86.00)	73.80	0.077
RS-13 Resilience Male (normal value: men aged 45–54)^◇^	437; 75.82 ± 16.90; 80.00 (74.00; 86.00)	75.00	0.314

Diamond ◇ = normal distribution; representation: n = number (percentage); mean ± standard deviation; median (1st quartile; 3rd quartile).

A clinically relevant impairment according to the criteria defined in the methods section was found in 0.51% (*n* = 6) in physical quality of life and 5.62% (*n* = 66) in mental quality of life, with more women affected (women 6.90% vs. men 3.44%; *P* = 0.013). The proportion of donors with elevated fatigue scores ranged from 4.69% for physical fatigue to 7.21% for mental fatigue. The mental fatigue scale showed the only gender difference, with more women affected (women 8.61% vs. men 4.79%; *P* = 0.015). Impairment on all five MFI scales was observed in 0.76% (*n* = 9) without gender-specific differences. Relevant depressive symptoms were observed in 0.75% (*n* = 9), relevant anxiety symptoms in 1.17% (*n* = 14), and relevant somatic symptom severity also in 1.17% (*n* = 14) of donors.

There was no gender difference in the resilience scale (Table 5). The values for both genders are comparable to the standard values for 45–54-year-olds (Table 6). Donors were less affected by psychosocial stressors (PHQ stress module) than the general population (*P* < 0.001), with women showing higher stress scores (*P* < 0.001). In the ambivalence scale, women showed less ambivalence than men (*P* = 0.003).

The regression analysis (Supplementary Tables S2A–E and Supplementary Figures S2A–E) revealed a lower mental quality of life in younger-aged donors (*P* < 0.001), females (*P* = 0.001), donors receiving psychotherapy (*P* < 0.001), donors burdened by caring for the recipient (*P* < 0.001), those with a higher PHQ stress score (*P* < 0.001), a lower resilience score (*P* = 0.004), and a higher ambivalence score (*P* < 0.001). The PHQ stress score and resilience score were also significantly associated with all fatigue scales (all *P* < 0.001). The ambivalence score, psychotherapy, and burden caused by caring for the recipient were significantly associated with three of the five fatigue scales. Gender did not show a significant correlation with any of the fatigue scales.

## Discussion

SOLKID-GNR prospectively records the somatic and mental health as well as the psychosocial situation of living donors in Germany with the aim of optimizing the selection process and aftercare. For the first time, the registry also enabled a representative proportion of living donors in Germany to be examined allowing a comprehensive description of the donor collective prior to donation and highlight its unique characteristics.

As in other countries, Germany has a higher proportion of female donors (29). The reasons for this gender imbalance are not fully understood. More men suffer from dialysis-requiring kidney disease and cardiovascular risk factors and are less likely to be considered as donors, presumably due to their more frequent unhealthy lifestyle (30, 31). Notably, SOLKID-GNR only records those who have been approved for donation. Furthermore, men are often the main income producers for the family, so their inability to work would be problematic for the family's economic situation, or at least more difficult to manage. This is consistent with our observation that male donors are more likely to be employed full-time than female donors. However, it should be noted that 77% of the female donors work at least part-time. It is also known that women are more likely to display relationship-oriented prosocial behavior (32, 33), see themselves more as helpers, and take on more caregiving responsibilities than men (34). In SOLKID-GNR, more women felt burdened by caring for the recipient. Nevertheless, they more often spontaneously decided to make a living donation and reported less ambivalence. More women stated that the initiative to donate came from themselves, but they also felt more often that they were “somewhat” pressured to donate. Thus, though debatable, women may experience greater social pressure to donate and feel more responsible for the recipient's wellbeing (35).

The low proportion of respondents who stated that the suggestion to donate came from medical professionals may be explained in part by the high standard of education and prior knowledge of donors. Further clearly interesting data for transplant centers is that a large proportion of donors felt that the preparation time for donation was excessively long.

The proportion of living donors of non-European origin in our cohort is very low. One reason why only 3% of living donors in the cohort were of non-European origin is that the self-assessment questionnaires currently available require knowledge of either German, Russian, or Turkish. About 9% of donors had to be excluded due to non-sufficient language skills, but not all of them were permanent residents of Germany. Further possible reasons for the low numbers may be a lower willingness to donate because of language barriers, limited access to understandable information as well as concerns about health restrictions and loss of employment.

Literature shows low living donation rates of families with a migrant background (36). Although the German National Transplant Registry records medical data on many recipients, migration-related variables (e.g., origin, ethnicity) are not collected and published in a standardized manner (37). There is currently no clear evidence that people with a migration background in Germany are generally overrepresented on transplant waiting lists. Very limited data available suggests that they are roughly proportional to their share of the population (38).

Comparing the available demographic data with earlier studies shows a gender-independent increase in the age of living donors over the past decades (6, 39, 40). Studies from the United States (14, 15) and Canada (16) found an average donor age at least 10 years younger than the German cohort. In a Norwegian study (17), donors were on average 8 years younger. The best match in terms of average age can be found in the Swiss National Registry with a mean age of 53 years (18).

Most donors described their general state of health as good. Nevertheless, the collective of living kidney donors in Germany by no means consists solely of healthy individuals (39). More than half had a pre-existing medical condition and/or were taking medication. Gender-specific differences showed that men had more cardiovascular risk factors: obesity (BMI > 30), hyperlipidaemia, hypertension, and active smoking status. Even in the Swiss living donor cohort the proportion of hypertensive living donor candidates requiring drug treatment was lower compared to the Germany cohort (18).

Living kidney donors accepted in Germany were found to have normal kidney function. Older donors predominantly have a creatinine clearance of less than 80 ml/min/1.73 m^2^, but the use of this threshold value for donation is controversial. British guidelines recommend an age- and gender-adjusted GFR threshold for acceptance (41, 42), whereas German transplant centers currently consider an eGFR < 80 ml/min/1.73 m^2^ to be a relative contraindication to donation (43). This limit also corresponds to the British guidelines on the accepted age-adjusted GFR for the current donor cohort, whose average age is 55. However, there is no uniform European standard for donor selection practices (44).

The donors' quality of life prior to living donation can be considered very good compared to the general population, and as evidenced by the low number of clinically relevant impaired individuals. The same applies to the psychological symptom scales and fatigue scales, among which mental fatigue had the largest proportion of impaired individuals, at 7.21%. The fact that only 0.76% showed impairment on all MFI scales suggests that fatigue symptoms affecting all areas of functioning are extremely rare prior to living donation. More women than men reported impairment in their mental quality of life and mental fatigue. Yet, the fact that women report worse scores than men in questionnaires on psychological wellbeing is a well-known phenomenon that is reflected in the different norm values of the relevant questionnaires and has previously been observed in individual studies in the context of living donation (45, 46). In relation to other countries, donors' quality of life appears to be comparable, as most studies also report above-average scores prior to donation (18, 47–49).

Regression analyses for mental quality of life and fatigue scales revealed several scales to be associated with factors such as psychotherapy (as an indicator of a mental disorder currently requiring treatment), perceived burden of caring for the recipient, and high ambivalence regarding living donation. The most consistent correlations were found for resilience and the PHQ stress score (degree of impairment due to psychosocial stressors). It is understandable that low psychological resilience and a high level of stress in various areas of life can negatively impact mental quality of life. The fact that these correlations were also found for the fatigue scales suggests that they could be at least partly psychogenic in origin or persistence. Identified stressors may also be attributable to the recipient's illness and its effects on the donor. Special attention should be paid to affected individuals during evaluation and subsequent follow-up care to provide adequate support (50).

The data presented are subject to certain limitations, as this is a cross-sectional analysis that does not allow for causal interpretations and does not provide information on postoperative outcomes. The registry will be able to provide such data, but our aim in this paper was to provide a comprehensive analysis of the characteristics of a representative cohort of German living donors prior to donation, which has not yet been described in such detail. A further limitation is the lack of data regarding non-participants, particularly on those who could not be included due to language barriers. The range of questionnaires available in languages other than German needs to be expanded. Nevertheless, we were able to include a large proportion of German living donors from most German transplant centers, thus achieving a multicentre design. We present an in-depth analysis that encompasses both medical and psychosocial data and focuses on gender-specific aspects, which we believe deserve more attention because of the apparent gender imbalance among donors.

In conclusion, we were able to show that German living donors are not a completely healthy population and older compared to the majority of donors in other countries. However, regardless of pre-existing conditions, most donors feel healthy and unaffected in their quality of life prior to donation. Women donate more frequently and are more determined to donate, but they are also more burdened by the care of the recipients. Of the few clinically relevant impairments, more women show impairments in their mental quality of life and mental fatigue. Affected individuals should receive special attention during the evaluation. The German Living Donation Registry offers a unique opportunity to prospectively study the impact of living donation in order to optimize the selection and counseling of donors and minimize the risks of living donation. We believe the results of this German registry are also relevant for other countries, as the trend toward older and less healthy donors is expected to be a global phenomenon.

## Data Availability

The raw data supporting the conclusions of this article will be made available by the authors, without undue reservation.

## Supplementary material

Supplementary Figure S1 Participating transplant centers.

Supplementary Figure S2 **(A)** Predictive factors for impaired physical quality of life (SF-12, PCS) (linear regression) for total group; OR, Odds ratio; CI, Confidence interval. **(B)**. Predictive factors for impaired mental quality of life (SF-12, MCS) (linear regression) for total group; OR, Odds ratio; CI, Confidence interval. **(C)** Predictive factors for impaired general fatigue (MFI-20) (linear regression) for total group; OR, Odds ratio; CI, Confidence interval. **(D)** Predictive factors for impaired physical fatigue (MFI-20) (linear regression) for total group; OR, Odds ratio; CI, Confidence interval. **(E)** Predictive factors for impaired mental fatigue (MFI-20) (linear regression) for total group; OR, Odds ratio; CI, Confidence interval. **(F)** Predictive factors for an eGFR ≤ 80 ml/min/1.73 m^2^ (logistic regression) for total group; OR, Odds ratio; CI, Confidence interval. **(G)** Predictive factors for an eGFR ≤ 80 ml/min/1.73 m^2^ (logistic regression) for women; OR, Odds ratio; CI, Confidence interval. **(H)** Predictive factors for an eGFR ≤ 80 ml/min/1.73 m^2^ (logistic regression) for men; OR, Odds ratio; CI, Confidence interval.

Supplementary material S1 Author List Registry Group.

Supplementary Table S1 Predictive factors for an eGFR ≤ 80 ml/min/1.73 m^2^ (logistic regression).

Supplementary Table S2 **(A–E)** Predictive factors for impaired quality of life and fatigue (linear regression).
