# Supplementary material for: Health status of living kidney donors and attitude toward donation–Results from the German Living Donor Registry (SOLKID-GNR)
Source: Front Med (Lausanne). 2026 Jun 10;13:1781270. doi: 10.3389/fmed.2026.1781270 (PMC13290515; doi:10.3389/fmed.2026.1781270)
Supplement: Supplementary Figure S1 — Participating transplant centers. [file Supplementary_file_1.zip › Supplementary Files/Suppl. Material S1.DOCX]

**Supplementary Material S1:**

**Author List Registry Group (in alphabetical order of the transplantation center)**

**Anja Mühlfeld,** MD, Division of Nephrology and Immunology, University Hospital RWTH Aachen, Aachen, Germany;

**Florian Sommer,** MD, Klinik für Allgemein-, Viszeral- und Transplantationschirurgie – Transplantationszentrum, Universitätsklinikum Augsburg, Augsburg, Germany;

**Aydin Er,** MD, Medizinische Klinik – Transplantationszentrum, Universitätsklinikum Augsburg, Augsburg, Germany;

**Klemens Budde,** MD, Department Nephrology and Medical Intensive Care, Charité University Berlin/Campus Mitte, Berlin, Germany;

**Lutz Liefeld**, MD, Department Nephrology and Medical Intensive Care, Charité University Berlin/Campus Mitte, Berlin, Germany;

**Fabian Halleck,** MD, Department Nephrology and Medical Intensive Care, Charité University Berlin/Campus Virchow, Berlin, Germany;

**Mira Choi**, MD, Department Nephrology and Medical Intensive Care, Charité University Berlin/Campus Virchow, Berlin, Germany;

**Julian Stumpf**, MD, University Hospital Carl Gustav Carus, TU Dresden, Dresden, Germany

**Juliane Putz**, MD, University Hospital Carl Gustav Carus, TU Dresden, Dresden, Germany

**Johannes Stegbauer,** MD, Department of Nephrology, Faculty of Medicine, University Hospital, Heinrich-Heine-University, Duesseldorf, Germany;

**Susanne Mende**, MD, Department of Nephrology, Faculty of Medicine, University Hospital, Heinrich-Heine-University, Duesseldorf, Germany;

**Mario Schiffer,** MD, Medizinische Klinik 4, Nephrologie und Hypertensiologie, Universitätsklinikum Erlangen, Erlangen, Germany;

**Katharina Heller**, MD, Medizinische Klinik 4, Nephrologie und Hypertensiologie, Transplantationszentrum, Universitätsklinikum Erlangen, Erlangen, Germany;

**Andreas Kribben**, MD, Klinik für Nephrologie, Universitätsklinikum Essen, Universität Duisburg-Essen, Essen, Germany;

**Bernd Jänigen,** MD, Department of General and Digestive Surgery, Section of Transplant Surgery, Faculty of Medicine, University of Freiburg, Freiburg, Germany;

**Johanna Schneider**, MD, Department of Medicine IV, University Freiburg Medical Center, Faculty of Medicine, University of Freiburg, Freiburg, Germany;

**Peter Benöhr,** MD, Medizinische Klinik III-Nephrologie, Klinikum Fulda gAG, Fulda, Germany

**Marion Haubitz**, MD, Medizinische Klinik III-Nephrologie, Klinikum Fulda gAG, Fulda, Germany

**Rolf Weimer**, MD, Department of Internal Medicine, Nephrology and Renal Transplantation, University Clinic of Giessen and Marburg (UKGM), Campus Giessen, Giessen, Germany;

**Hristos Karakizlis**, MD, Department of Internal Medicine, Nephrology and Renal Transplantation, University Clinic of Giessen and Marburg (UKGM), Campus Giessen, Giessen, Germany;

**Ulrich Pein**, MD, University Hospital Halle (Saale), Department of Internal Medicine, Halle (Saale), Germany;

**Lutz Fischer,** MD, Klinik und Poliklinik für Viszerale Transplantationschirurgie und Universitäres Transplantations Centrum, Uniklinikum Hamburg-Eppendorf, Hamburg, Germany;

**Florian Grahammer,** MD, III. Department of Medicine, University Hospital Hamburg-Eppendorf; Hamburg Center for Kidney Health, University Hospital Hamburg-Eppendorf; University Transplant Center, University Hospital Hamburg-Eppendorf, Hamburg, Germany;

**Peter Weithofer,** MD, Nephrologisches Zentrum Niedersachsen, Klinikum Hann. Münden GmbH, Hann. Münden, Germany;

**Volker Kliem,** MD, Nephrologisches Zentrum Niedersachsen, Klinikum Hann. Münden GmbH, Hann. Münden, Germany;

**Martin Zeier,** MD, Department of Nephrology, University Hospital Heidelberg, Heidelberg, Germany; **Mandy Schlosser,** MD, Klinik für Innere Medizin III, Universitätsklinikum Jena, Jena, Germany;

**Gunter Wolf**, MD, Klinik für Innere Medizin III, Universitätsklinikum Jena, Jena, Germany;

**Thomas Rath,** MD, Abteilung für Nephrologie und Transplantationsmedizin, Westpfalz-Klinikum Kaiserslautern, Kaiserslautern, Germany;

**Christian Mönch**, MD, Klinik für Allgemein-, Viszeral-, Kinder- und Transplantationschirurgie, Westpfalz-Klinikum Kaiserslautern, Kaiserslautern, Germany;

**Kevin Schulte,** MD, Department of Nephrology and Hypertension, University Hospital Schleswig-Holstein, Kiel, Germany;

**Friedrich A. von Samson-Himmelstjerna**, MD, Department of Nephrology and Hypertension, University Hospital Schleswig-Holstein, Kiel, Germany;

**Dirk Stippel,** MD, Department of General, Visceral, Cancer and Transplant Surgery, University of Cologne, Faculty of Medicine and University Hospital Cologne, Cologne, Germany;

**Christine Kurschat,** MD, Department II of Internal Medicine and Center for Molecular Medicine Cologne, University of Cologne, Faculty of Medicine and University Hospital Cologne, Cologne, Germany;

**Ana Harth,** MD, Medizinische Klinik I (Klinik für Nephrologie, Transplantationsmedizin und internistische Intensivmedizin), Kliniken der Stadt Köln, Cologne, Germany;

**Ruth Hackenberg,** MD, Medizinische Klinik I (Klinik für Nephrologie, Transplantationsmedizin und internistische Intensivmedizin), Kliniken der Stadt Köln, Cologne, Germany;

**Anette Bachmann,** MD, Department für Innere Medizin, Neurologie und Dermatologie, Klinik für Endokrinologie/Nephrologie, Universitätsklinikum Leipzig, Leipzig, Germany;

**Antje Weimann**, MD, Department für Innere Medizin, Neurologie und Dermatologie, Klinik für Endokrinologie/Nephrologie, Universitätsklinikum Leipzig, Leipzig, Germany;

**Martin Nitschke,** MD, Medizinische Klinik I, Nephrologie Universitätsklinikum Schleswig-Holstein, Campus Lübeck, Lübeck, Germany;

**Figen Cakiroglu**, MD, Medizinische Klinik I, Nephrologie Universitätsklinikum Schleswig-Holstein, Campus Lübeck, Lübeck, Germany;

**Julia Weinmann-Menke**, MD, I. Medizinische Klinik und Poliklinik, Universitätsmedizin Mainz, Mainz, Germany;

**Birgit Kortus-Götze,** MD, Klinik für Innere Medizin, Nephrologie, Universitätsklinikum Gießen und Marburg, Standort Marburg, Marburg, Germany;

**Joachim Hoyer**, MD, Klinik für Innere Medizin, Nephrologie, Universitätsklinikum Gießen und Marburg, Standort Marburg, Marburg, Germany;

**Stephan Kemmner**, MD, Transplantationszentrum, Klinikum der Universität München, Munich, Germany;

**Manfred Stangl**, MD, Klinik für Allgemeine, Viszeral-, Transplantations-, Gefäß- und Thoraxchirurgie, Klinikum der Universität München, Campus Großhadern, Munich, Germany;

**Lutz Renders**, MD, Abteilung für Nephrologie, TUM Universitätsklinikum, Klinikum rechts der Isar, Munich, Germany;

**Volker Assfalg**, MD, Klinik und Poliklinik für Chirurgie, TUM Universitätsklinikum, Klinikum rechts der Isar, Munich, Germany;

**Stefan Reuter**, MD, Department of Internal Medicine, Transplant Nephrology, University Hospital Muenster, Muenster, Germany;

**Daniel Zecher**, MD, Department of Nephrology, University Hospital Regensburg, Regensburg, Germany;

**Christina Hackl,** MD, Department of Surgery, University Hospital Regensburg, Regensburg, Germany; **Vedat Schwenger,** MD, Klinik für Nieren-, Hochdruck- und Autoimmunerkrankungen, Klinikum der Landeshauptstadt Stuttgart gKAöR, Katharinenhospital, Stuttgart, Germany;

**Markus Krautter**, MD, Klinik für Nieren-, Hochdruck- und Autoimmunerkrankungen, Klinikum der Landeshauptstadt Stuttgart gKAöR, Katharinenhospital, Stuttgart, Germany;

**Anja Schork,** MD, Department of Diabetology, Endocrinology, Nephrology, Section of Nephrology and Hypertension, University of Tübingen, Tübingen, Germany

**Silvio Nadalin**, MD, Department of General Visceral and Transplant Surgery, University of Tübingen, Tübingen, Germany;

**Kai Lopau,** MD, Department of Nephrology, University Hospital Wuerzburg, Wuerzburg, Germany;

**Anna Laura Herzog**, MD, Transplantation Center, University Hospital Wuerzburg, Wuerzburg, Germany
