# Supplementary material for: Health status of living kidney donors and attitude toward donation–Results from the German Living Donor Registry (SOLKID-GNR)
Source: Front Med (Lausanne). 2026 Jun 10;13:1781270. doi: 10.3389/fmed.2026.1781270 (PMC13290515; doi:10.3389/fmed.2026.1781270)
Supplement: Supplementary Figure S1 — Participating transplant centers. [file Supplementary_file_1.zip › Supplementary Files/Suppl. Table S1.DOCX]

**Supplementary Table S1: Predictive factors for an eGFR ≤ 80 ml/min/1.73 m² (logistic regression)**

| Parameter | Total | | | | Women | | | | Men | | | |
| --- | --- | --- | --- | --- | --- | --- | --- | --- | --- | --- | --- | --- |
| Effect | Odds Ratio | 95% Wald Confidence Limits | | P-Value | Odds Ratio | 95% Wald Confidence Limits | | P-Value | Odds Ratio | 95% Wald Confidence Limits | | P-Value |
| Age | 1.07 | 1.05 | 1.10 | <0.001 | 0.95 | 0.92 | 0.97 | <0.001 | 1.11 | 1.06 | 1.15 | <0.001 |
| Smoking status (2 categories): Active/former vs. Never smoked (Ref.) | 0.96 | 0.64 | 1.44 | 0.843 | 0.80 | 0.49 | 1.32 | 0.387 | 0.69 | 0.36 | 1.34 | 0.269 |
| Previous cardiovascular disease: Yes vs. No (Ref.) | 1.29 | 0.57 | 2.94 | 0.539 | 0.85 | 0.31 | 2.38 | 0.761 | 1.15 | 0.28 | 4.64 | 0.849 |
| BMI ≥ 30: Yes vs. No (Ref.) | 1.04 | 0.58 | 1.87 | 0.903 | 0.96 | 0.46 | 2.01 | 0.917 | 0.86 | 0.32 | 2.35 | 0.774 |
| Previous tumour disease: Yes vs. No (Ref.) | 1.46 | 0.60 | 3.58 | 0.408 | 0.42 | 0.15 | 1.15 | 0.091 | 0.61 | 0.12 | 3.06 | 0.544 |
| Diabetes medication: Yes vs. No (Ref.) | 1.32 | 0.15 | 11.64 | 0.801 | 0.44 | 0.03 | 6.32 | 0.545 |  |  |  |  |
| High blood pressure medication: Yes vs. No (Ref.) | 0.79 | 0.50 | 1.25 | 0.314 | 1.35 | 0.75 | 2.41 | 0.319 | 0.87 | 0.42 | 1.80 | 0.714 |
| Albumin/creatinine ratio: cutoff >30 mg/g vs. ≤30 mg/g (Ref.) | 0.19 | 0.03 | 1.47 | 0.111 | 3.44 | 0.44 | 26.88 | 0.239 |  |  |  |  |
| HbA1c: cutoff ≥6% vs. <6% (Ref.) | 2.00 | 0.92 | 4.33 | 0.080 | 0.46 | 0.17 | 1.29 | 0.141 | 1.60 | 0.45 | 5.64 | 0.465 |
| Mean Arterial Pressure (MAP) | 0.99 | 0.97 | 1.01 | 0.289 | 1.01 | 0.99 | 1.03 | 0.413 | 0.98 | 0.95 | 1.02 | 0.339 |

(Ref.)=Reference category; BMI=Body Mass Index
