# Supplementary material for: Health status of living kidney donors and attitude toward donation–Results from the German Living Donor Registry (SOLKID-GNR)
Source: Front Med (Lausanne). 2026 Jun 10;13:1781270. doi: 10.3389/fmed.2026.1781270 (PMC13290515; doi:10.3389/fmed.2026.1781270)
Supplement: Supplementary Figure S1 — Participating transplant centers. [file Supplementary_file_1.zip › Supplementary Files/Suppl. Tables S2A-E.DOCX]

**Supplementary Tables 2 A-E: Predictive factors for impaired quality of life and fatigue (linear regression)**

**A)**

| Physical quality of life (SF-12 PCS) | | | | |
| --- | --- | --- | --- | --- |
| Parameters | Estimate | 95% Confidence Limits | | P-Value |
| Intercept | 57.62 | 56.20 | 59.03 |  |
| Burden on family carers: Not affected vs. Slightly affected (Ref.) | 0.22 | -0.40 | 0.85 | 0.702 |
| Burden on family carers: Severely affected vs. Slightly affected (Ref.) | -0.29 | -1.87 | 1.29 |  |
| PHQ stress score | -0.05 | -0.16 | 0.07 | 0.443 |
| Age | -0.05 | -0.07 | -0.03 | <0.001 |
| Burden of caring for the recipient: Not at all vs. Very severe to Somewhat (Ref.) | 0.27 | -0.17 | 0.72 | 0.232 |
| Other care-dependent persons: Yes vs. No (Ref.) | -0.08 | -0.68 | 0.53 | 0.803 |
| Gender: Male vs. Female (Ref.) | -0.26 | -0.68 | 0.15 | 0.213 |
| Previous malignant disease: Yes vs. No (Ref.) | -1.02 | -2.02 | -0.03 | 0.043 |
| Chronic pain disorder: Yes vs. No (Ref.) | -3.88 | -4.97 | -2.80 | <0.001 |
| Previous cardiovascular disease: Yes vs. No (Ref.) | -1.54 | -2.47 | -0.60 | 0.001 |
| Previous immunological disease: Yes vs. No (Ref.) | 0.18 | -0.73 | 1.08 | 0.699 |
| Taking psychiatric medication: Yes vs. No (Ref.) | -2.30 | -3.96 | -0.64 | 0.007 |
| Psychotherapeutic treatment: Yes vs. No (Ref.) | 0.48 | -0.75 | 1.71 | 0.442 |
| Previous mental illness: Yes vs. No (Ref.) | -0.88 | -1.93 | 0.16 | 0.099 |

(Ref.)=Reference category

**B)**

| Mental quality of life (SF-12 MCS) | | | | |
| --- | --- | --- | --- | --- |
| Parameter | Estimate | 95% Confidence Limits | | P-Value |
| Intercept | 50.32 | 47.44 | 53.21 |  |
| Burden on family carers: Not affected vs. Slightly affected (Ref.) | 0.13 | -0.88 | 1.15 | 0.414 |
| Burden on family carers: Severely affected vs. Slightly affected (Ref.) | -1.67 | -4.30 | 0.95 |  |
| PHQ stress score | -1.20 | -1.39 | -1.00 | <0.001 |
| Age | 0.07 | 0.03 | 0.10 | <0.001 |
| Burden of caring for the recipient: Not at all vs. Very severe to Somewhat (Ref.) | 1.34 | 0.63 | 2.05 | <0.001 |
| Other persons dependent on care: Yes vs. No (Ref.) | 0.40 | -0.57 | 1.36 | 0.417 |
| Ambivalence scale | -0.58 | -0.83 | -0.34 | <0.001 |
| Gender: Male vs. Female (Ref.) | 1.13 | 0.46 | 1.81 | 0.001 |
| Previous malignancy: Yes vs. No (Ref.) | 0.72 | -0.88 | 2.31 | 0.378 |
| School leaving certificate: General higher education entrance qualification (*Abitur*) vs. Primary school/Secondary school/Polytechnic secondary school (9th grade) (Ref.) | 0.49 | -0.48 | 1.46 | 0.556 |
| School leaving certificate: Other school leaving certificate vs. primary school/secondary school/polytechnic secondary school (9th grade) (Ref.) | -0.09 | -2.53 | 2.34 |  |
| School leaving certificate: Technical college entrance qualification (*Fachabitur*) vs. primary school/secondary school/polytechnic secondary school (9th grade) (Ref.) | 0.13 | -1.05 | 1.31 |  |
| School leaving certificate: No school leaving certificate vs. primary school/secondary school/polytechnic secondary school (9th grade) (Ref.) | 2.13 | -0.34 | 4.60 |  |
| School leaving certificate: Secondary school leaving certificate (*mittlere Reife*)/polytechnic secondary school (10th grade) vs. primary school/secondary school/polytechnic secondary school (9th grade) (Ref.) | 0.50 | -0.38 | 1.38 |  |
| Committed relationship: Yes vs. No (Ref.) | -0.34 | -1.32 | 0.63 | 0.492 |
| Chronic pain condition: Yes vs. No (Ref.) | 1.09 | -0.66 | 2.83 | 0.221 |
| Resilience scale (RS-13) | 0.03 | 0.01 | 0.04 | 0.004 |
| Taking psychiatric medication: Yes vs. No (Ref.) | 1.57 | -1.09 | 4.22 | 0.247 |
| Psychotherapeutic treatment: Yes vs. No (Ref.) | -5.66 | -7.64 | -3.68 | <0.001 |
| Previous mental illness: Yes vs. No (Ref.) | -1.41 | -3.08 | 0.27 | 0.101 |

(Ref.)=Reference category

**C)**

| General Fatigue (MFI-20) | | | | |
| --- | --- | --- | --- | --- |
| Parameter | Estimate | 95% Confidence Limits | | P-Value |
| Intercept | 7.76 | 6.42 | 9.11 |  |
| Burden on family carers: Not affected vs. Slightly affected (Ref.) | 0.02 | -0.45 | 0.50 | 0.524 |
| Burden on family carers: Severely affected vs. Slightly affected (Ref.) | -0.69 | -1.92 | 0.53 |  |
| PHQ stress score | 0.57 | 0.48 | 0.66 | <0.001 |
| Age | -0.01 | -0.02 | 0.01 | 0.428 |
| Burden of caring for the recipient: Not at all vs. Very severe to Somewhat (Ref.) | -0.48 | -0.81 | -0.14 | 0.005 |
| Other persons dependent on care: Yes vs. No (Ref.) | 0.30 | -0.06 | 0.28 | 0.190 |
| Ambivalence scale | 0.17 | -0.37 | 0.26 | 0.003 |
| Gender: Male vs. Female (Ref.) | -0.06 | -0.89 | 0.61 | 0.718 |
| Previous malignancy: Yes vs. No (Ref.) | -0.14 | -0.89 | 0.61 | 0.714 |
| Chronic pain disorder: Yes vs. No (Ref.) | 0.07 | -0.73 | 0.88 | 0.859 |
| School leaving certificate: General higher education entrance qualification (*Abitur*) vs. Primary school/secondary school/polytechnic secondary school (9th grade) (Ref.) | 0.03 | -0.42 | 0.48 | 0.855 |
| School leaving certificate: Other school leaving certificate vs. Primary school/secondary school/polytechnic secondary school (9th grade) (Ref.) | 0.02 | -1.10 | 1.13 |  |
| School leaving certificate: Technical college entrance qualification (*Fachabitur*) vs. Primary school/secondary school/polytechnic secondary school (9th grade) (Ref.) | 0.13 | -0.42 | 0.68 |  |
| School leaving certificate: No school leaving certificate vs. Primary school/secondary school/polytechnic secondary school (9th grade) (Ref.) | -0.69 | -1.85 | 0.46 |  |
| School leaving certificate: Secondary school leaving certificate (intermediate school leaving certificate)/polytechnic secondary school (10th grade) vs. Primary school/secondary school/polytechnic secondary school (9th grade) (Ref.) | 0.07 | -0.34 | 0.48 |  |
| Committed relationship: Yes vs. No (Ref.) | 0.12 | -0.33 | 0.58 | 0.601 |
| Resilience scale (RS-13) | -0.02 | -0.03 | -0.01 | <0.001 |
| Taking psychiatric medications: Yes vs. No (Ref.) | 1.20 | -0.04 | 2.45 | 0.058 |
| Psychotherapeutic treatment: Yes vs. No (Ref.) | 0.72 | -0.21 | 1.65 | 0.127 |
| Previous mental illness: Yes vs. No (Ref.) | 0.48 | -0.30 | 1.27 | 0.228 |

(Ref.)= Reference category

**D)**

| Physical Fatigue (MFI-20) | | | | |
| --- | --- | --- | --- | --- |
| Parameter | Estimate | 95% Confidence Limits | | P-Value |
| Intercept | 6.81 | 5.46 | 8.12 |  |
| Burden on family carers: Not affected vs. Slightly affected (Ref.) | -0.14 | -0.62 | 0.33 | 0.802 |
| Burden on family carers: Severely affected vs. Slightly affected (Ref.) | -0.24 | -1.47 | 0.99 |  |
| PHQ stress score | 0.39 | 0.30 | 0.48 | <0.001 |
| Age | 0.02 | <0.01 | 0.03 | 0.025 |
| Burden of caring for the recipient: Not at all vs. Very severe to Somewhat (Ref.) | -0.09 | -0.42 | 0.24 | 0.598 |
| Other persons dependent on care: Yes vs. No (Ref.) | 0.04 | -0.41 | 0.49 | 0.858 |
| Ambivalence scale | 0.04 | -0.07 | 0.15 | 0.499 |
| Gender: Male vs. Female (Ref.) | 0.03 | -0.29 | 0.34 | 0.871 |
| Previous malignancy: Yes vs. No (Ref.) | 0.27 | -0.48 | 1.02 | 0.477 |
| Chronic pain disorder: Yes vs. No (Ref.) | 0.52 | -0.29 | 1.33 | 0.212 |
| School leaving certificate: General higher education entrance qualification (*Abitur*) vs. Primary school/secondary school/polytechnic secondary school (9th grade) (Ref.) | -0.58 | -1.03 | -0.13 | 0.120 |
| School leaving certificate: Other school leaving certificate vs. Primary school/secondary school/polytechnic secondary school (9th grade) (Ref.) | 0.44 | -0.67 | 1.56 |  |
| School leaving certificate: Technical college entrance qualification (*Fachabitur*) vs. Primary school/secondary school/polytechnic secondary school (9th grade) (Ref.) | -0.40 | -0.95 | 0.15 |  |
| School leaving certificate: No school leaving certificate vs. Primary school/secondary school/polytechnic secondary school (9th grade) (Ref.) | -0.19 | -1.34 | 0.97 |  |
| School leaving certificate: Secondary school leaving certificate (intermediate school leaving certificate)/polytechnic secondary school (10th grade) vs. Primary school/secondary school/polytechnic secondary school (9th grade) (Ref.) | -0.21 | -0.62 | 0.20 |  |
| Committed relationship: Yes vs. No (Ref.) | -0.01 | -0.46 | 0.44 | 0.965 |
| Resilience scale (RS-13) | -0.02 | -0.03 | -0.01 | <0.001 |
| Taking psychiatric medication: Yes vs. No (Ref.) | 0.38 | -0.87 | 1.63 | 0.551 |
| Psychotherapeutic treatment: Yes vs. No (Ref.) | 1.07 | 0.14 | 2.00 | 0.024 |
| Previous mental illness: Yes vs. No (Ref.) | 0.31 | -0.49 | 1.11 | 0.444 |

(Ref.)= Reference category

**E)**

| Mental Fatigue (MFI-20) | | | | |
| --- | --- | --- | --- | --- |
| Parameter | Estimate | 95% Confidence Limits | | P-Value |
| Intercept | 8.45 | 7.11 | 9.80 |  |
| Burden on family carers: Not affected vs. Slightly affected (Ref.) | 0.12 | -0.35 | 0.59 | 0.435 |
| Burden on family carers: Severely affected vs. Slightly affected (Ref.) | -0.69 | -1.91 | 0.54 |  |
| PHQ stress score | 0.54 | 0.45 | 0.63 | <0.001 |
| Age | -0.01 | -0.03 | <0.01 | 0.106 |
| Burden of caring for the recipients: Not at all vs. Very severe to Somewhat (Ref.) | -0.48 | -0.81 | -0.15 | 0.004 |
| Other persons dependent on care: Yes vs. No (Ref.) | -0.60 | -1.05 | -0.15 | 0.009 |
| Ambivalence scale | 0.16 | 0.04 | 0.27 | 0.007 |
| Gender: Male vs. Female (Ref.) | -0.14 | -0.45 | 0.17 | 0.385 |
| Previous malignancy: Yes vs. No (Ref.) | -0.35 | -1.09 | 0.40 | 0.361 |
| Chronic pain disorder: Yes vs. No (Ref.) | -0.53 | -1.34 | 0.27 | 0.195 |
| School leaving certificate: General higher education entrance qualification (*Abitur*) vs. Primary school/secondary school/polytechnic secondary school (9th grade) (Ref.) | -0.28 | -0.72 | 0.17 | 0.349 |
| School leaving certificate: Other school leaving certificate vs. Primary school/secondary school/polytechnic secondary school (9th grade) (Ref.) | -0.54 | -1.65 | 0.57 |  |
| School leaving certificate: Technical college entrance qualification (*Fachabitur*) vs. Primary school/secondary school/polytechnic secondary school (9th grade) (Ref.) | -0.09 | -0.63 | 0.46 |  |
| School leaving certificate: No school leaving certificate vs. Primary school/secondary school/polytechnic secondary school (9th grade) (Ref.) | -0.85 | -2.00 | 0.30 |  |
| School leaving certificate: Secondary school leaving certificate (intermediate school leaving certificate)/polytechnic secondary school (10th grade) vs. Primary school/secondary school/polytechnic secondary school (9th grade) (Ref.) | -0.39 | -0.80 | 0.01 |  |
| Committed relationship: Yes vs. No (Ref.) | 0.41 | -0.04 | 0.87 | 0.074 |
| Resilience scale (RS-13) | -0.03 | -0.04 | -0.02 | <0.001 |
| Taking psychiatric medication: Yes vs. No (Ref.) | 0.10 | -1.14 | 1.34 | 0.879 |
| Psychotherapeutic treatment: Yes vs. No (Ref.) | 2.01 | 1.08 | 2.94 | <0.001 |
| Previous mental illness: Yes vs. No (Ref.) | 0.76 | -0.02 | 1.54 | 0.057 |

(Ref.)= Reference category
