# Supplementary figures and images for: Health status of living kidney donors and attitude toward donation–Results from the German Living Donor Registry (SOLKID-GNR)
Source: Front Med (Lausanne). 2026 Jun 10;13:1781270. doi: 10.3389/fmed.2026.1781270 (PMC13290515; doi:10.3389/fmed.2026.1781270)

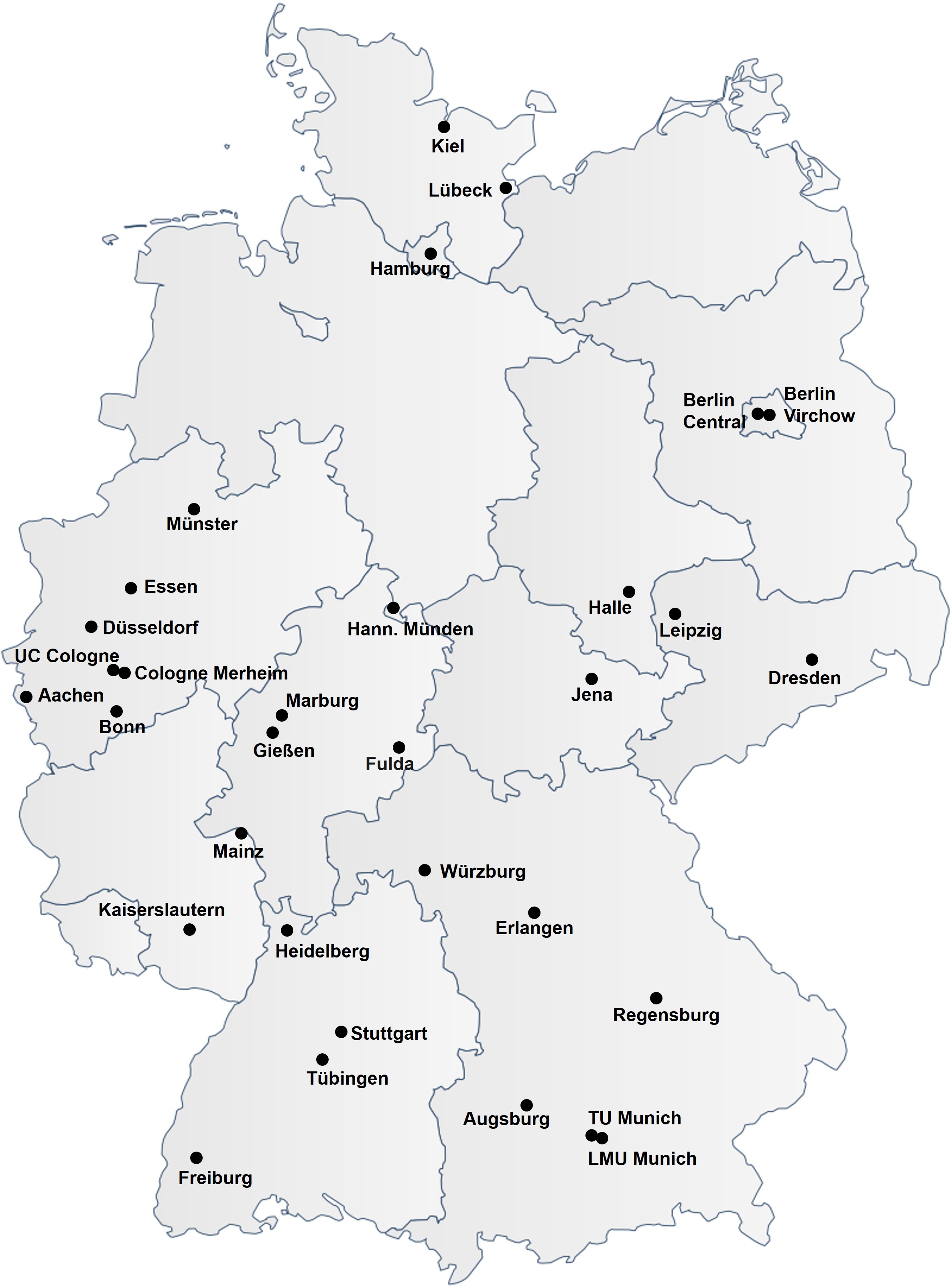

Supplement: Supplementary Figure S1 — Participating transplant centers. [file Supplementary_file_1.zip › Supplementary Files/Suppl. Figure S1.JPEG]

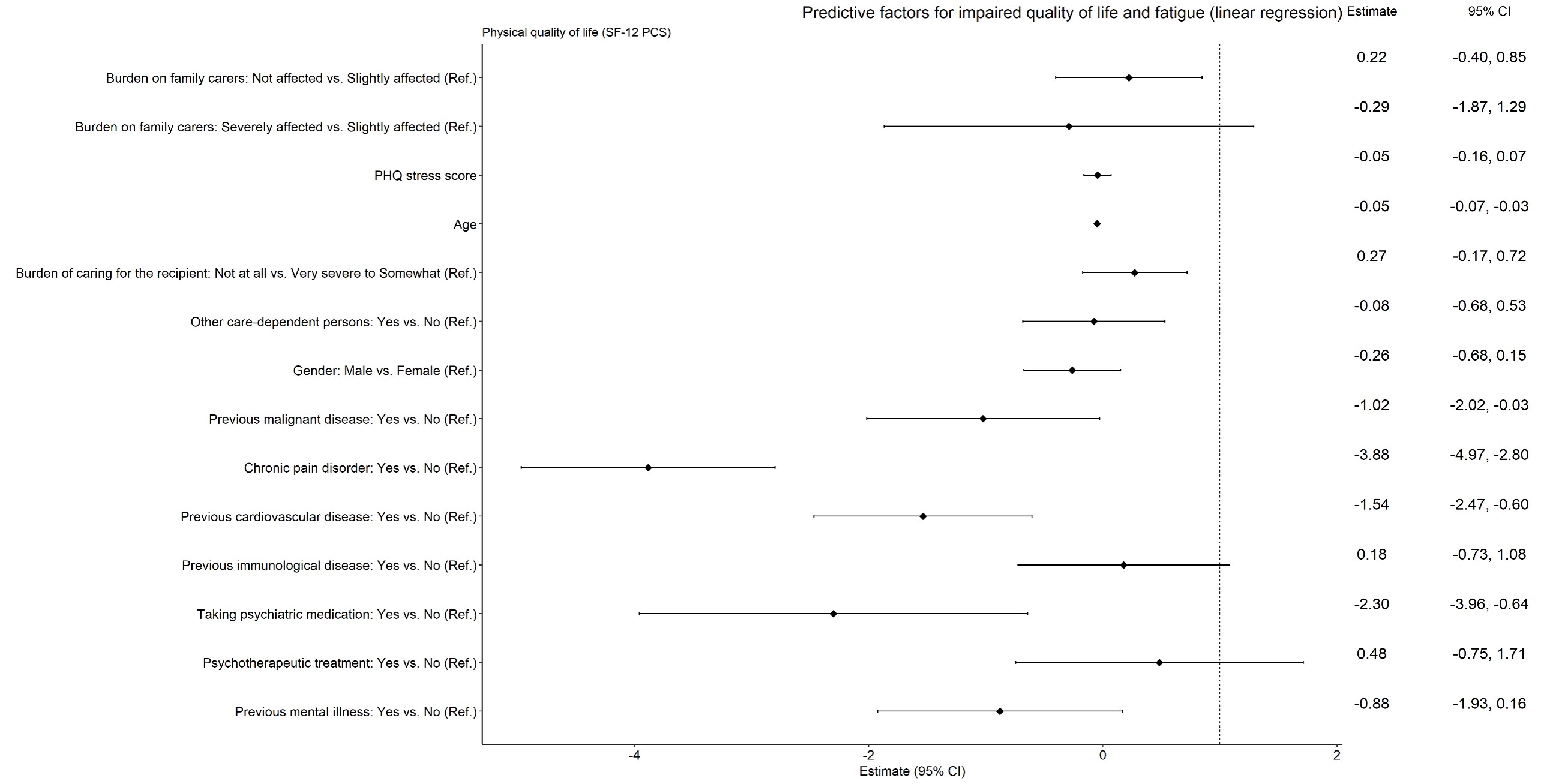

Supplement: Supplementary Figure S1 — Participating transplant centers. [file Supplementary_file_1.zip › Supplementary Files/Suppl. Figure S2A.JPEG]

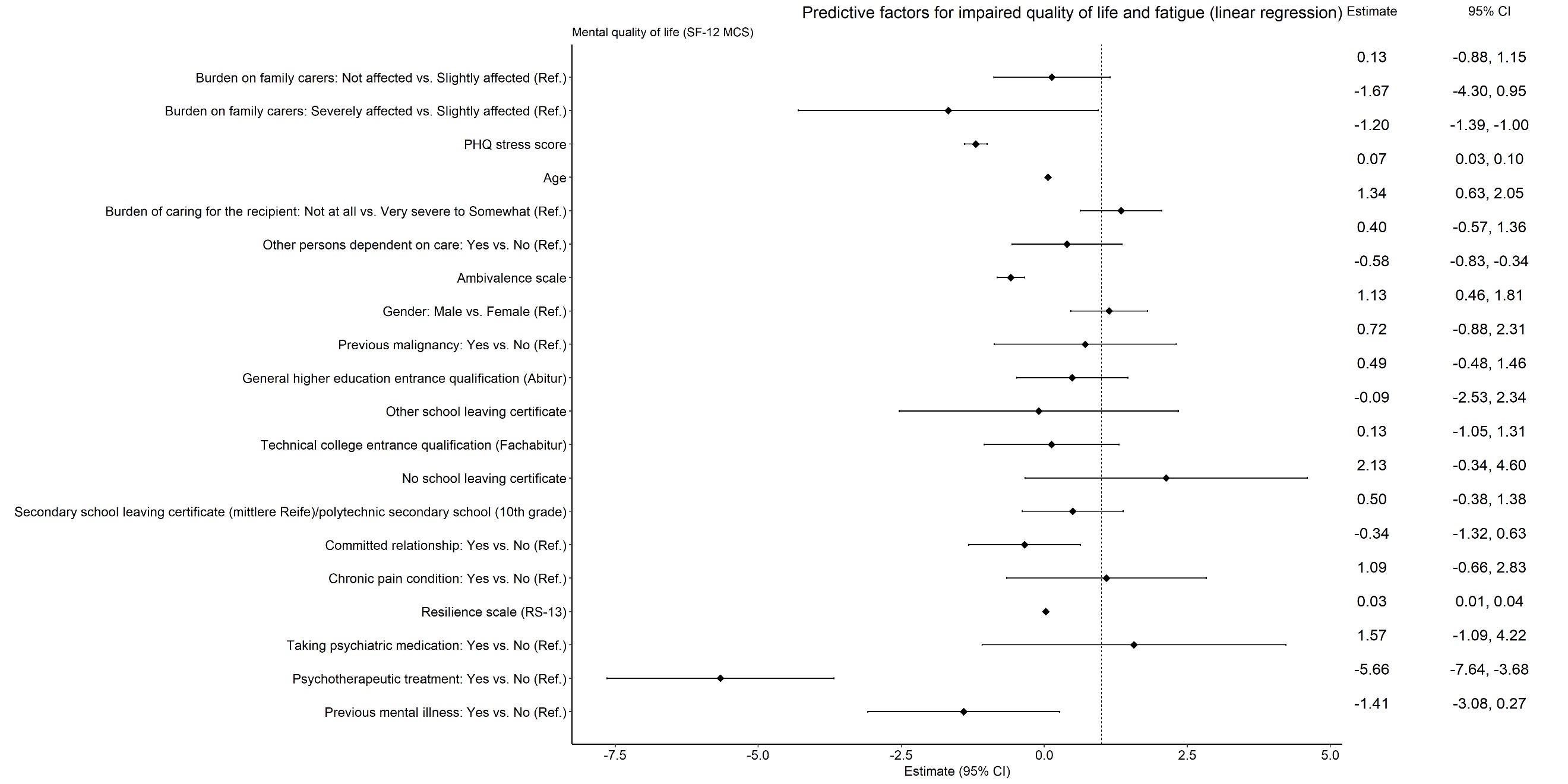

Supplement: Supplementary Figure S1 — Participating transplant centers. [file Supplementary_file_1.zip › Supplementary Files/Suppl. Figure S2B.JPEG]

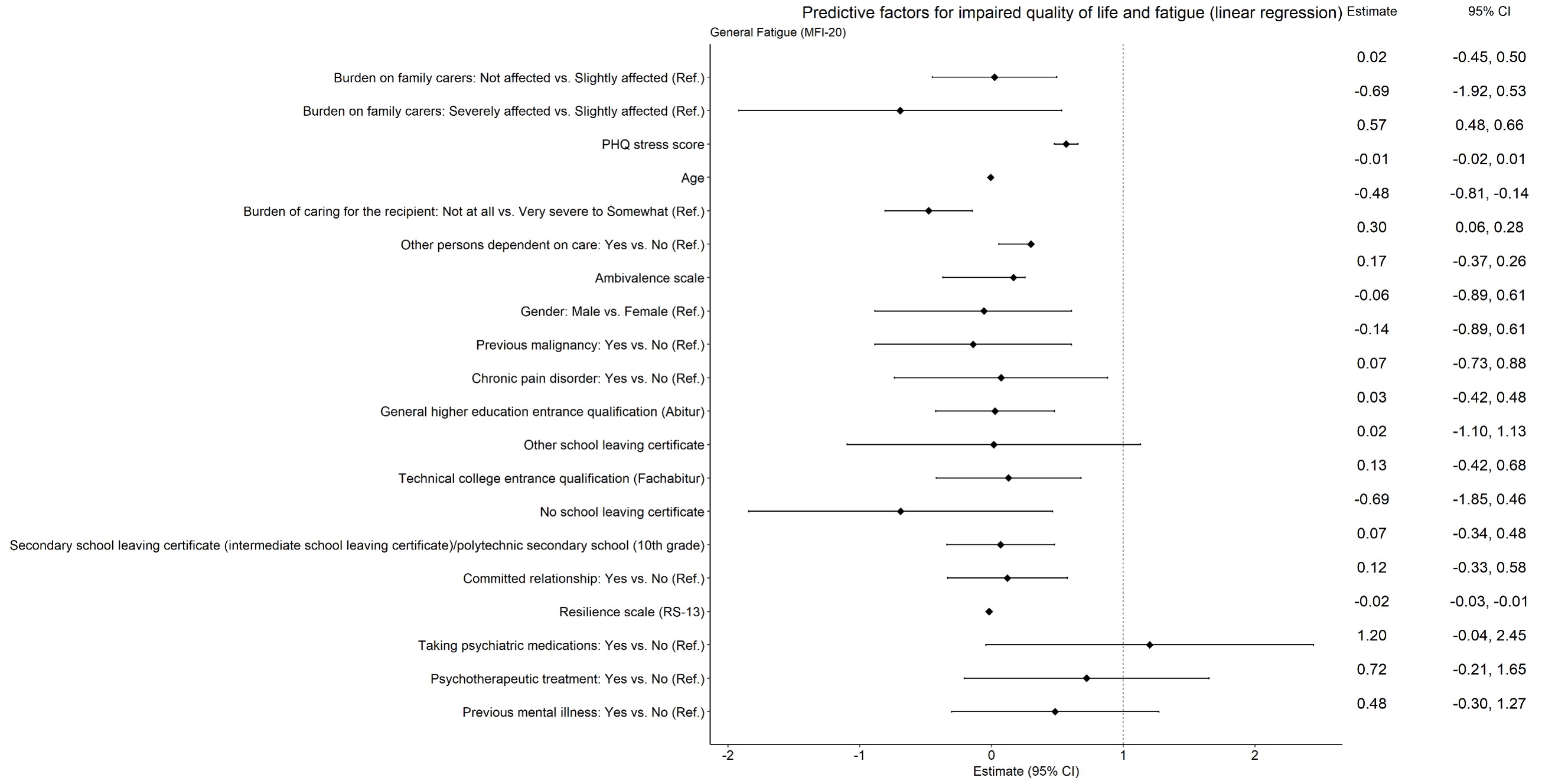

Supplement: Supplementary Figure S1 — Participating transplant centers. [file Supplementary_file_1.zip › Supplementary Files/Suppl. Figure S2C.JPEG]

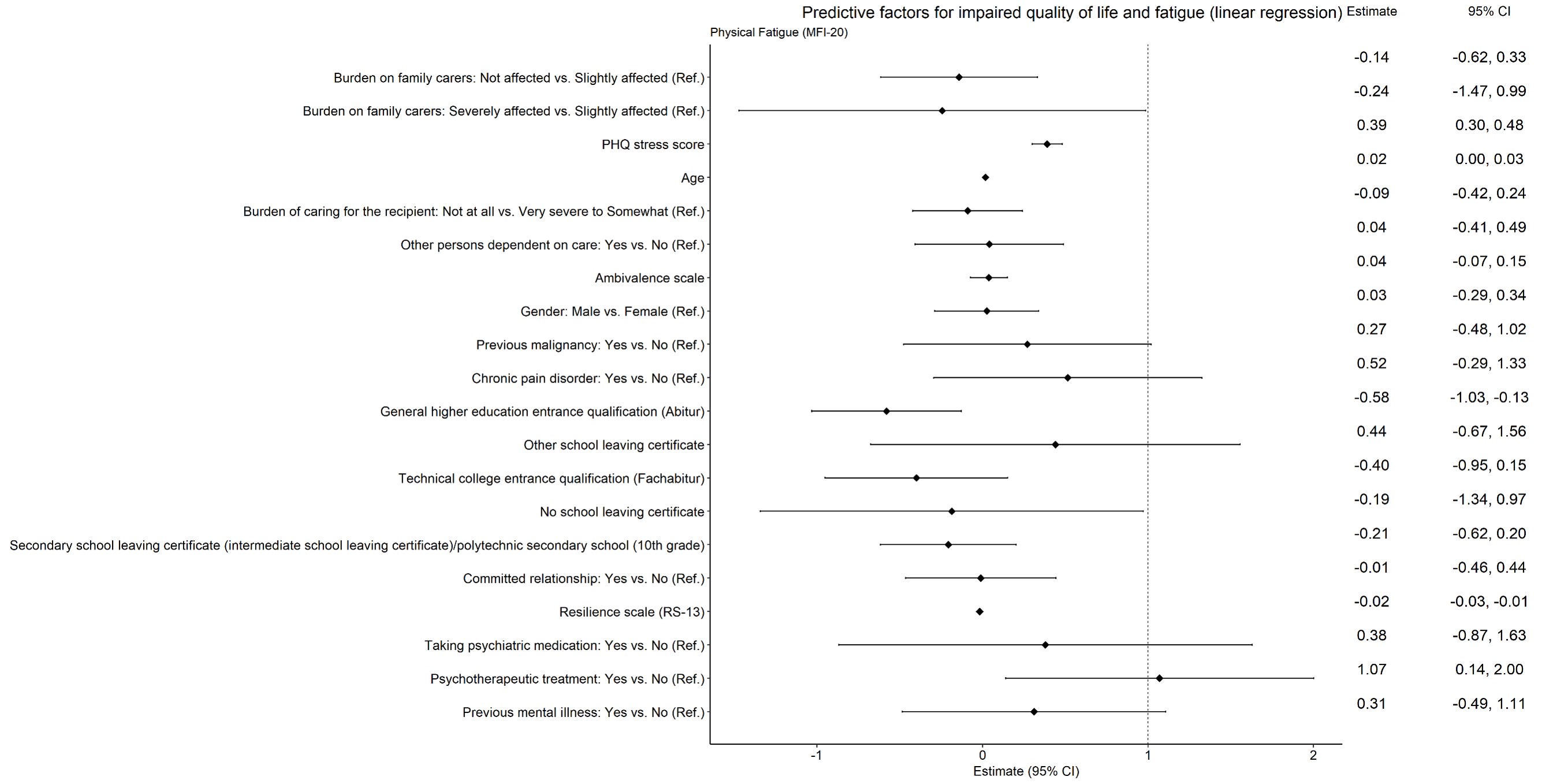

Supplement: Supplementary Figure S1 — Participating transplant centers. [file Supplementary_file_1.zip › Supplementary Files/Suppl. Figure S2D.JPEG]

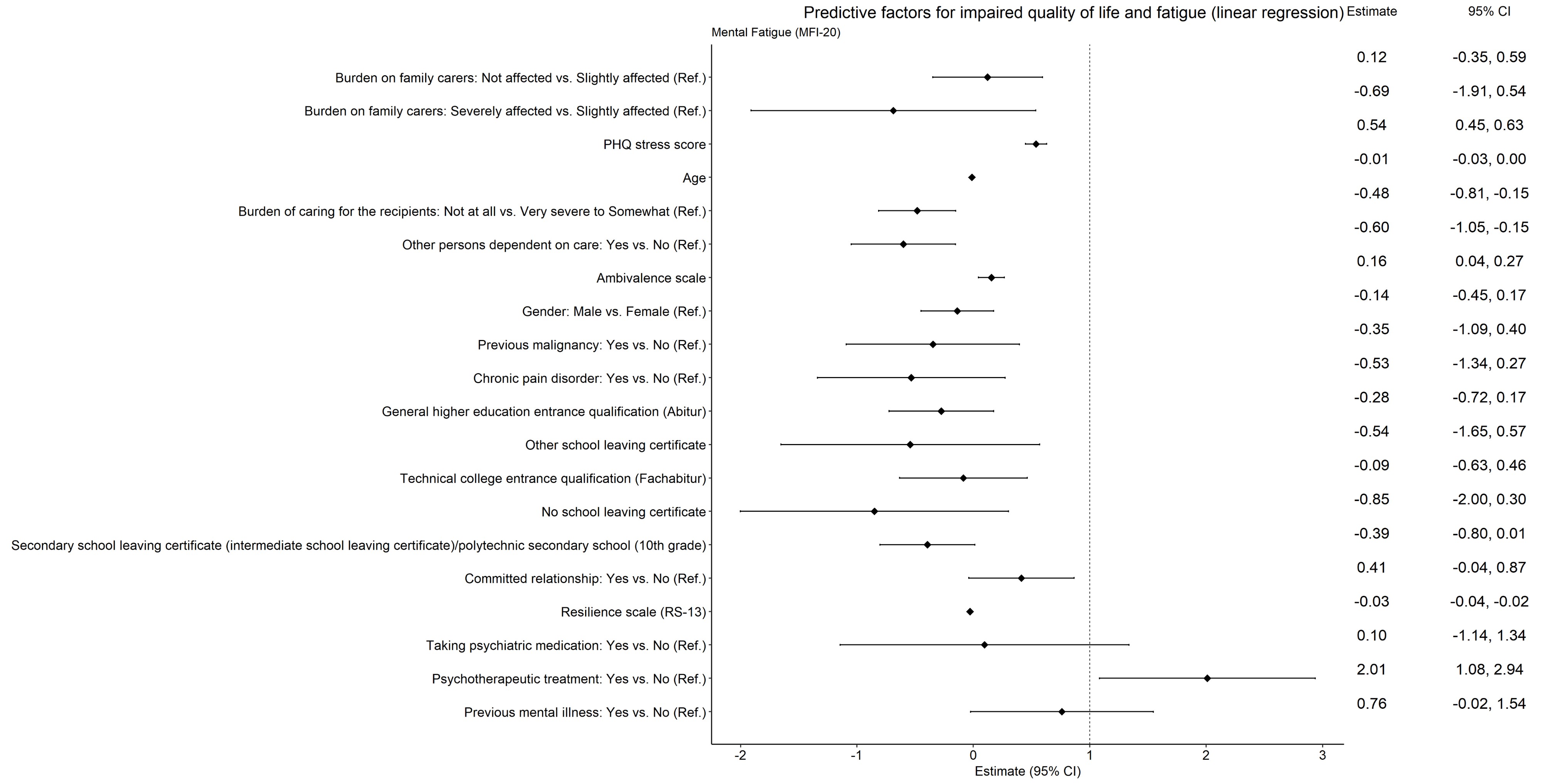

Supplement: Supplementary Figure S1 — Participating transplant centers. [file Supplementary_file_1.zip › Supplementary Files/Suppl. Figure S2E.JPEG]

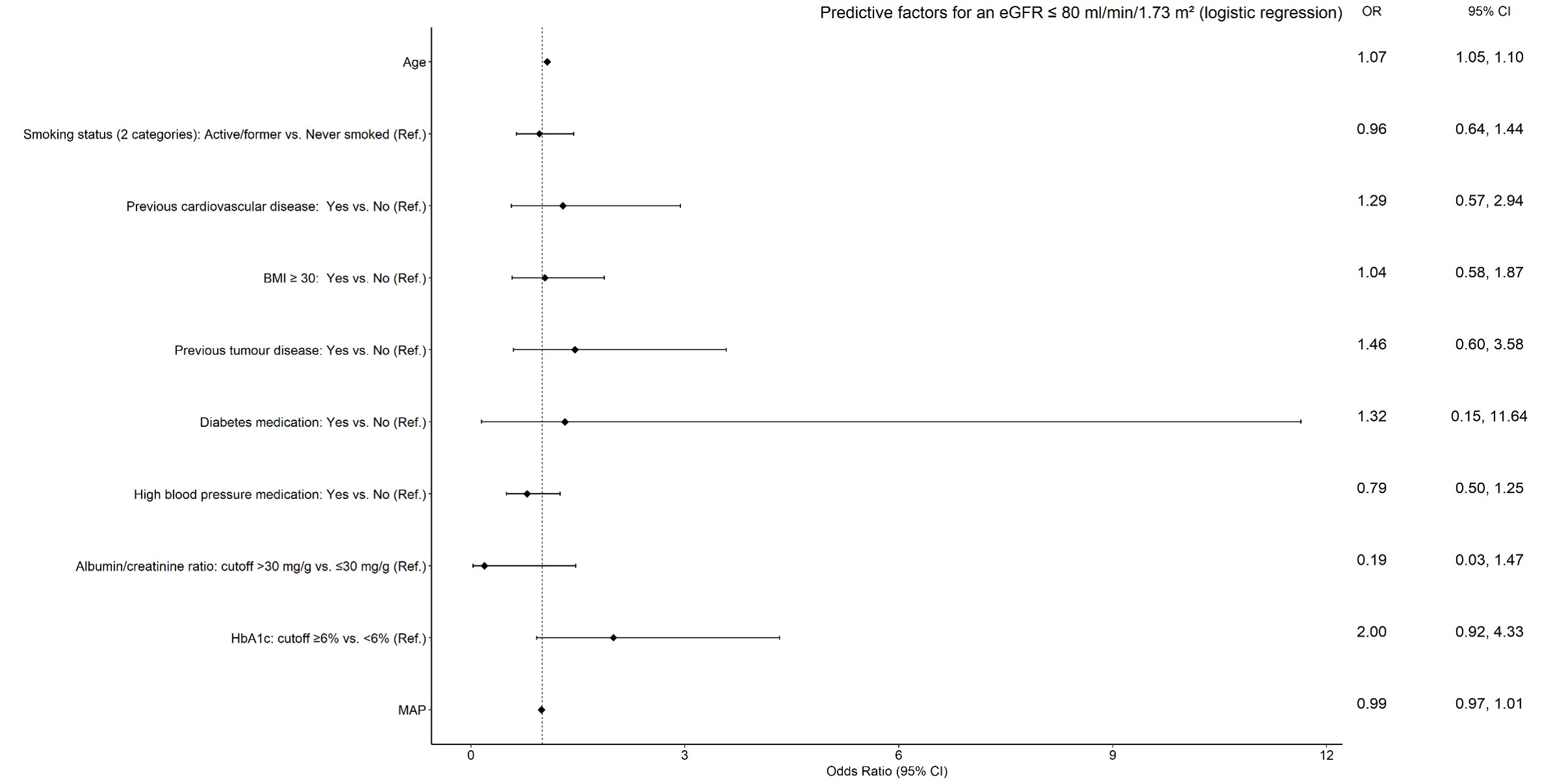

Supplement: Supplementary Figure S1 — Participating transplant centers. [file Supplementary_file_1.zip › Supplementary Files/Suppl. Figure S2F.JPEG]

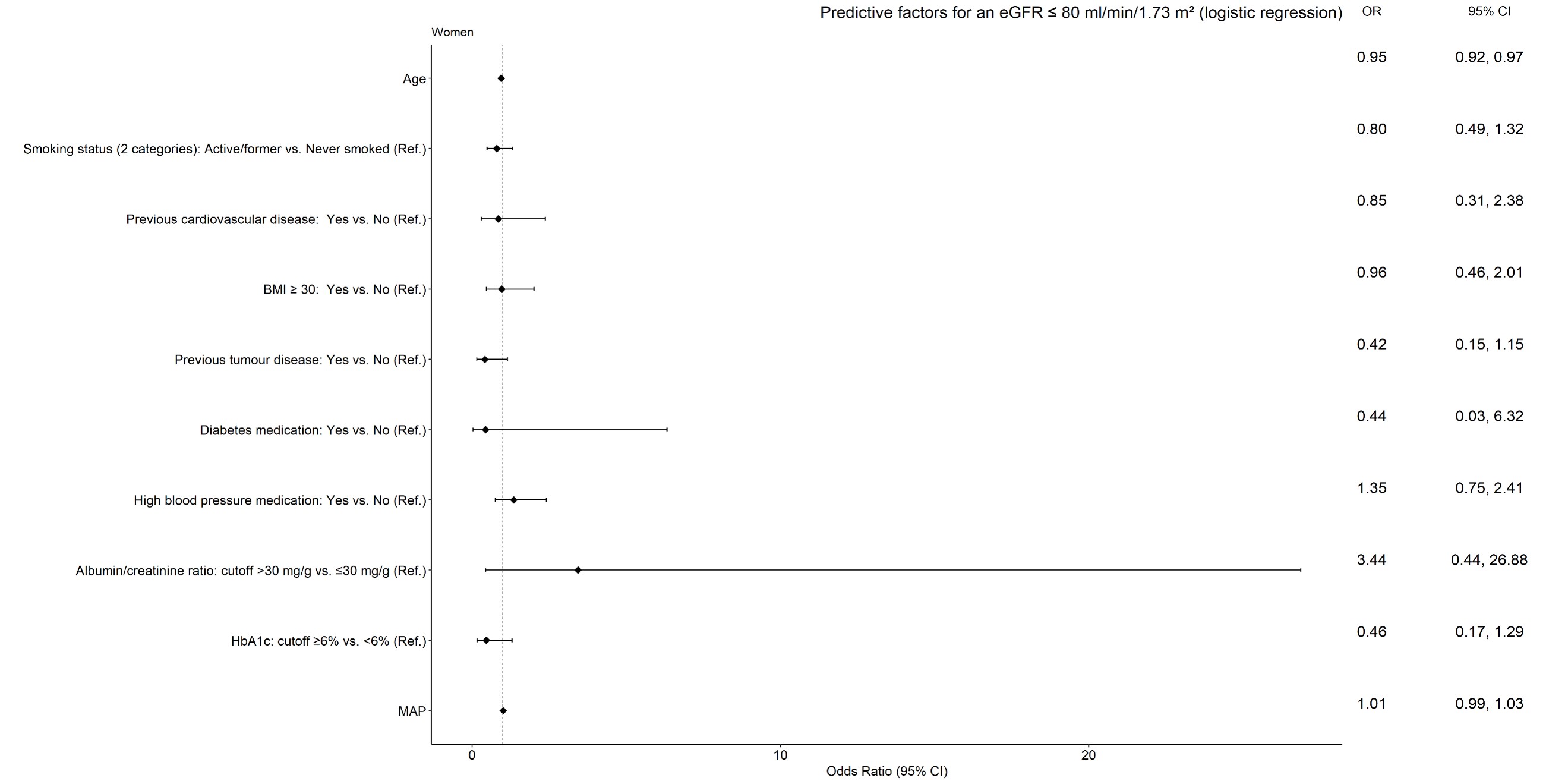

Supplement: Supplementary Figure S1 — Participating transplant centers. [file Supplementary_file_1.zip › Supplementary Files/Suppl. Figure S2G.JPEG]

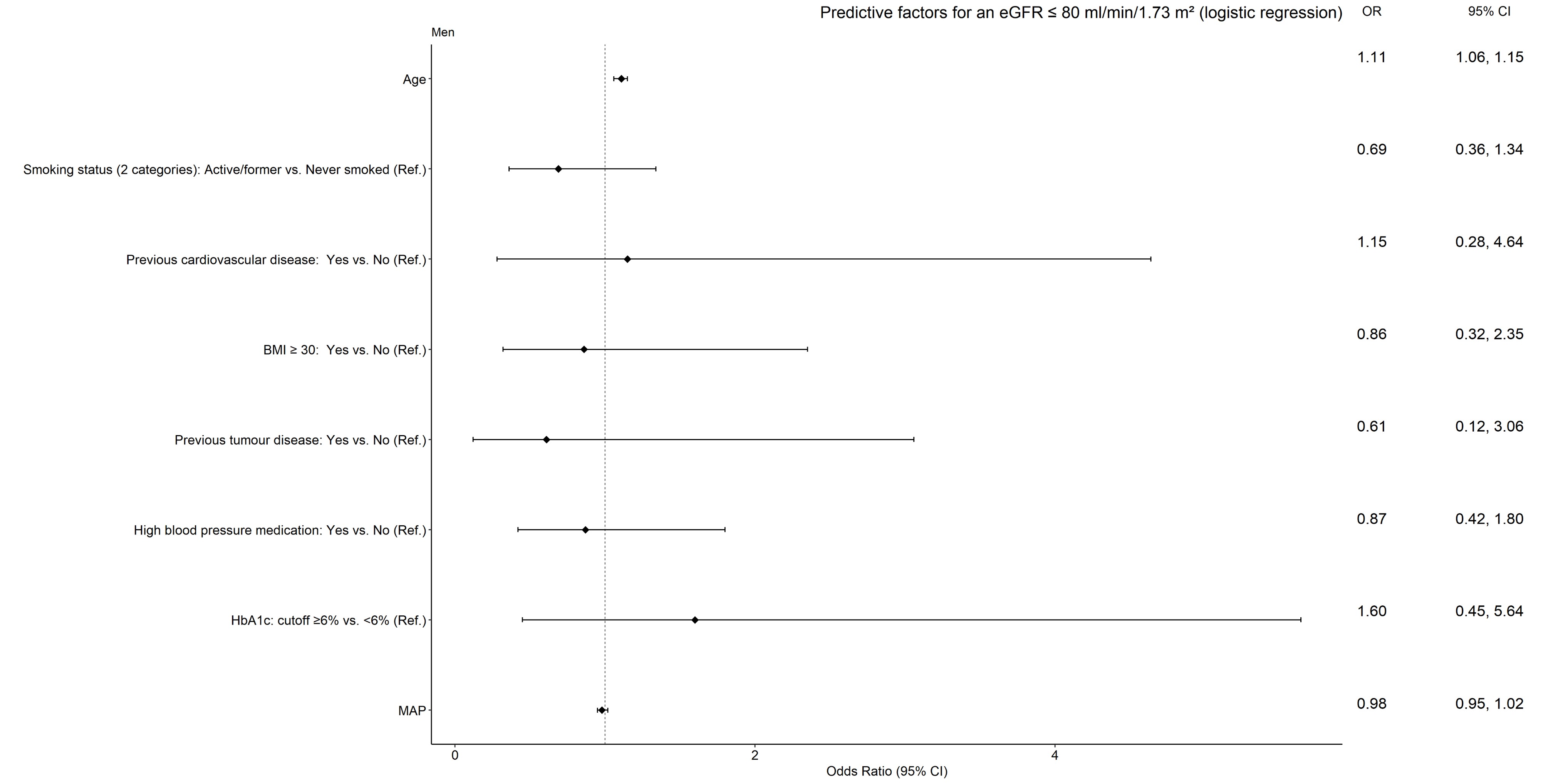

Supplement: Supplementary Figure S1 — Participating transplant centers. [file Supplementary_file_1.zip › Supplementary Files/Suppl. Figure S2H.JPEG]
